# The Orphan Response Regulator Aor1 Is a New Relevant Piece in the Complex Puzzle of *Streptomyces coelicolor* Antibiotic Regulatory Network

**DOI:** 10.3389/fmicb.2017.02444

**Published:** 2017-12-12

**Authors:** Sergio Antoraz, Sergio Rico, Héctor Rodríguez, Laura Sevillano, Juan F. Alzate, Ramón I. Santamaría, Margarita Díaz

**Affiliations:** ^1^Departamento de Microbiología y Genética, Instituto de Biología Funcional y Genómica, Consejo Superior de Investigaciones Científicas, Universidad de Salamanca, Salamanca, Spain; ^2^Cic bioGUNE, Derio, Spain; ^3^Departamento de Microbiología y Parasitología, Facultad de Medicina, Centro Nacional de Secuenciación Genómica, Sede de Investigación Universitaria, Universidad de Antioquia, Medellín, Colombia

**Keywords:** *Streptomyces*, two-component systems, antibiotic production, orphan response regulator, crosstalk, stress response

## Abstract

*Streptomyces coelicolor*, the best-known biological antibiotic producer, encodes 29 predicted orphan response regulators (RR) with a putative role in the response to environmental stimuli. However, their implication in relation to secondary metabolite production is mostly unexplored. Here, we show how the deletion of the orphan RR Aor1 (SCO2281) provoked a drastic decrease in the production of the three main antibiotics produced by *S. coelicolor* and a delay in morphological differentiation. With the aim to better understand the transcriptional events underpinning these phenotypes, and the global role of Aor1 in *Streptomyces*, a transcriptional fingerprint of the Δ*aor1* mutant was compared to a wild-type strain. RNA-Seq analysis revealed that the deletion of this orphan regulator affects a strikingly high number of genes, such as the genes involved in secondary metabolism, which matches the antibiotic production profiles observed. Of particular note, the sigma factor SigB and all of the genes comprising its regulon were up regulated in the mutant. Our results show that this event links osmotic stress to secondary metabolite production in *S. coelicolor* and indicates that the RR encoded by *aor1* could be a key regulator in both of these processes.

## Introduction

The ubiquity of the genus *Streptomyces*, comprised of Gram-positive filamentous bacteria, has greatly facilitated its presence as a normal inhabitant of soil, marine niches, and also as a symbiotic partner of sponges, plants, and a plethora of other types of organisms (Chankhamhaengdecha et al., 2013; Braña et al., 2015). All species of this genus harbor the genetic information to produce around 20–30 different secondary metabolites, such as antibiotics (Chaudhary et al., 2013), which in nature may be used as signals in the intra- and interspecies communication that establishes relationships among the organisms within a community (symbiotic, antagonistic, etc.) (Vetsigian et al., 2011; Diminic et al., 2014). The production of secondary metabolites takes place as a response to the different physical (pH, temperature, pressure, etc.) and chemical (nutrients, elicitors, etc.) signals present in the environment through complex regulatory networks (Liu et al., 2013). The wide range of environmental and physiological responses to stimuli generated by bacteria can be appreciated within the database of the Prokaryotic 2-Component Systems (P2CS^1^). Two-component systems (TCSs) are able to recognize change, and integrate this information to elaborate a proper response, which includes the production of antibiotics.

A model of a typical TCS consists of a membrane-bound Histidine Kinase (HK) that acts as a sensor and is auto-phosphorylated at a specific histidine residue upon receiving a stimulus. Later on, the phosphoryl group is transferred to an aspartic acid of the cytoplasmic Response Regulator (RR), which triggers its activation through a conformational change. Finally, this protein completes the response by mainly regulating the expression of target genes. Both HK and RR genes usually lie in close proximity to one another on a chromosome and form an operon. However, there are also a considerable number of orphans, genes that lack a counterpart in the genome, which ultimately broadens the response capacity of cells (Schaller et al., 2011).

Three main mechanisms ensure partner specificity in bacteria: molecular recognition, phosphatase activity, and substrate competition (Podgornaia and Laub, 2013). A small number of residues are responsible for maintaining the specific molecular recognition in protein–protein interaction interfaces between HKs and RRs (Capra et al., 2010). Furthermore, the HK may act as both a kinase and a phosphatase in these systems. The phosphatase reaction serves, in part, to modulate the level of pathway output and to inhibit the pathway after an activating signal has subsided (Podgornaia and Laub, 2013). Crosstalk between different pathways, leading to the activation of a RR by a non-cognate HK or by promiscuous phosphodonors, such as acetyl-phosphate, can also be avoided by the phosphatase activity of the cognate HK (Salazar and Laub, 2015). Nevertheless, the existence of different inner checkpoints for maintaining fidelity does not imply that such TCSs systems cannot play a role in the activity of more than one interacting partner. However, although adding crosstalk in TCSs may have disastrous consequences for cell fitness, it is important for the evolution of new TCS pathways after the duplication of an existing pathway being immediately unavoidable the crosstalk (Capra et al., 2012; Rowland and Deeds, 2014).

The number of TCSs present in an organism is directly correlated to its habitat (Schaller et al., 2011) and can be variable: zero in *Mycoplasma genitalium*; 30 HKs and 32 RRs in *Escherichia coli*; 36 HKs and 34 RRs in *Bacillus subtilis*; up to 131 HKs and 80 RRs in *Anabaena* sp.; and 132 HKs and 119 RRs in *Myxococcus xanthus* (Jung et al., 2012). This evolutionary advantage may allow an organism to respond quickly to all possible situations.

Many *Streptomyces* genomes have been sequenced to date, and more than 50 TCSs and approximately 20 orphan HKs and 20 orphan RRs (P2CS database) have been identified in all of them. This fact reinforces the idea that *Streptomyces* requires a high number of these TCSs for surviving in a changeable and hostile environment and to cope with a complex lifestyle. The present study has been carried out using *Streptomyces coelicolor*, whose genome contains 100 HKs, 87 RRs, 20 orphan HKs and 29 RRs (P2CS database) (Bentley et al., 2002). However, only 17 TCSs have so far been studied, and most of these systems are, at least partially, involved in antibiotic biosynthesis (Rodríguez et al., 2013).

One of the TCS previously described by our group, AbrC (*SCO4596/97/98*) (Yepes et al., 2011; Rico et al., 2014a; Rodríguez et al., 2015), positively regulates the production of antibiotics. Wang et al. reported that AbrC3 (SCO4596) has a high amino acid sequence identity (59%) with the orphan RR SCO2281, where the conserved identity among most RRs is around 30% (Wang et al., 2009). These authors propose that such highly conserved RRs could be regulated by the same HK, which may suggest possible crosstalk between HK AbrC2 (SCO4597) and the RR SCO2281 (Wang et al., 2009). Moreover, the pleiotropic phenotypes of the triple mutant of the AbrC system and the single Δ*abrC3* mutant also suggest crosstalk between AbrC HKs and other RRs (Rico et al., 2014a).

In this work we describe for the first time, the involvement of the orphan RR SCO2281 in the regulation of antibiotic production. This gene has been named *aor1* from *a*ntibiotic *o*rphan *r*egulator, which we will use from now on. Here, we show how the deletion of this gene is characterized by a drastic decrease in the production of the three main antibiotics produced by *S. coelicolor* (actinorhodin – ACT, undecylprodiginine – RED, and calcium dependent antibiotic – CDA), and also by a delay in morphological differentiation, suggesting that Aor1, like AbrC3, acts as a positive regulator of both processes. Our RNA-Seq results show that Aor1 plays a crucial role in a gene regulation cascade of *S. coelicolor* probably integrating a response to different signals, and establishing a connection between secondary metabolism (through several regulators) and the response to osmotic stress (mediated partially by SigB) within a high level of the regulatory network.

## Materials and Methods

### Strains, Media and Growth Conditions

*Escherichia coli* strain BW25113 (pIJ790) (containing the λRed system) (Datsenko and Wanner, 2000) and non-methylating ET12567 (pUZ8002) (harboring the *tra* genes in the non-transmissible RP4-derivative plasmid pUZ8002) (MacNeil et al., 1992) were used for PCR-targeted mutagenesis of *S. coelicolor* M145. *E. coli* DH5α and ET12567 (a dam^-^ strain) were used to obtain the DNA to transform *S. coelicolor*. For CDA bioassays, a parent strain of *Bacillus subtilis* (CECT 4522) was grown as an overlay on NA medium (Hopwood et al., 1985). *S. coelicolor* M145 (prototroph; SCP1^-^, SCP2^-^) and the Δ*aor1* mutant strain were grown on R2YE, MSA, YEPD, NMMP, PGA, R5 and LB solid media for transformation, sporulation, spore quantification, and phenotypic assays (Fernández et al., 1998; Kieser et al., 2000). LB medium (Sambrook et al., 1989) was used for RNA-Seq experiment. When necessary, the medium was supplemented with antibiotics: *E. coli* media - ampicillin (100 μg mL^-1^), apramycin (50 μg mL^-1^), kanamycin (50 μg mL^-1^), chloramphenicol (25 μg mL^-1^), or nalidixic acid (25 μg mL^-1^) and *S. coelicolor* media – neomycin (20 μg mL^-1^) or thiostrepton (10 μg mL^-1^).

### DNA Manipulation

Plasmid isolation, restriction enzyme digestion, ligation, and transformation of *E. coli* and *S. coelicolor* were carried out using the methods of Green and Sambrook (2012), and Kieser et al. (2000), respectively. The plasmids and cosmids used are listed in Supplementary Table S1.

### Mutant Construction

REDIRECT PCR-targeting technology was used to replace the coding region of the *SCO2281* gene with an apramycin resistance cassette [*aac(3)IV* gene] in cosmid SCC30 (Gust et al., 2003). The primers used to amplify the mutagenesis cassette (SRG-28 and SRG-30) with pIJ773 as the template are listed in Supplementary Table S2. The mutated cosmid SCC30 Δ*SCO2281::acc(3)IV* (ΔSCC30-1) obtained in *E. coli* BW25113(pIJ790) were demethylated in the ET12567(pUZ8002) strain and transferred by conjugation to *S. coelicolor* M145. The desired double recombinants carrying apramycin resistance and sensitive to kanamycin (the selection marker for the vector sequences) were selected. Southern blotting and PCR assays confirmed the deletion of the *SCO2281* gene in *S. coelicolor* M145.

### Antibiotic Production Analysis

ACT and RED antibiotic production was assayed on solid LB medium plates inoculated with 10^5^ spores added in a 5 μL drop at 30°C. RED production was detected after 2 days as the red color of colonies. ACT production was observed after 3 days of growth as a blue halo around the colonies. For CDA, after 2 days of growth on NA, the plates were overlaid with 5 mL of soft agar plus Ca(NO_3_)_2_ (70 mM) inoculated with *B. subtilis* as the test microorganism (0.2 mL, 0.25 DO) and incubated at 30°C for 24 h. A replica plate without calcium was used as a negative control. These experiments were all performed in triplicate.

Antibiotic production was assayed from cultures in liquid LB medium inoculated with 10^6^ spores mL^-1^ at 30°C. ACT and RED antibiotic production were quantified using the spectrophotometric method described in Yepes et al. (2011). For CDA, plates of NA with and without Ca(NO_3_)_2_ (70 mM) were inoculated on the surface with 200 μL of *B. subtilis* (DO_600_ 0.25) using sterile glass balls. Afterward, a sterile cork borer was used on these plates to make wells with a diameter 0.8 cm. 150 μL of the supernatant of a 48 h *Streptomyces* culture were added into the wells and incubated at 30°C for 24 h.

### Plasmid Construction

The integrative plasmid pSETaor1 (Supplementary Table S1) was constructed by cloning the *aor1* gene and its promoter region in the shuttle *Streptomyces* integrative plasmid pSET152t. First, the *aor1* sequence was cloned in the bifunctional *E. coli*–*Streptomyces* plasmid pXHis1 in the NdeI and XhoI sites, remaining under the control of the *xysA* promoter and yielding the intermediate plasmid pXHisaor1. Second, the PCR-amplified *aor1* promoter, cloned with the oligonucleotides SRG-37 and SRG-38 and the NdeI and EcoRI sites, replaced the *xysA* promoter, obtaining the pHisaor1 vector. This promoter corresponds to the 545 bp upstream region of *SCO2282* that forms an operon with *aor1* gene. Finally, the BglII–BglII fragment of pHisaor1, containing the *aor1* gene plus its promoter, was cloned into the previously de-phosphorylated BamHI site in pSET152t, obtaining the integrative plasmid pSETaor1.

The multicopy plasmid pNXaor1 was obtained by cloning the NdeI/HindIII fragment from the plasmid pXHisaor1 into the same sites of pNX24 (pN702GEM3 derivative). To construct the multicopy plasmid pNaor1, its own promoter (upstream region of *SCO2282*) previously amplified by PCR was cloned as an EcoRI/NdeI fragment into the same sites of pNXaor1 (Supplementary Table S1).

### qRT-PCR

Specific primers for the genes tested were designed using the Primer3 web-based tool^2^ (Supplementary Table S2). Five microgram of the RNA samples were treated with RNAase-free DNaseI (Promega) according to the manufacturer’s instructions. One microgram of the resulting RNA was used as template for cDNA synthesis using iScript Reverse Transcription Supermix for RT-qPCR (Bio-Rad) in 20 μL reaction volumes. The samples were diluted 1:1 with distilled water and 2 μL was used in the quantitative PCR reaction with 10 pmol of forward and reverse primer and 5 μL of SsoAdvanced SYBR Green Supermix (Bio-rad).

Each assay was performed in duplicate using the CFX96 Touch^TM^ Real-Time PCR Detection System (Bio-Rad). Control PCRs were included to check that there was neither DNA (without RT, enzyme negative control) nor environmental contamination. Absolute quantification was performed using decimal serial dilutions of *S. coelicolor* genomic DNA with a known number of copies as standard.

### Comparative Transcriptome Analysis

*Streptomyces coelicolor* M145 and *S. coelicolor* Δ*aor1* were grown in LB medium inoculated with 10^6^ sp mL^-1^ for 36 h at 28°C. The cells from three biological replicates were collected and treated with RNA protect, and afterward, RNA was extracted with RNeasy Mini Kit (Qiagen) following the company instructions. RNA samples quantity and quality were performed by Nanodrop and Bioanalyzer (Agilent). All samples passed the quality threshold of RIN > 7.

Macrogen Inc. (South Korea) provided the RNA-Seq data. The rRNA was removed with Ribo-Zero rRNA Removal Kit (Bacteria) and one library per sample was made using the Illumina TruSeq Stranded Total RNA kits. An independent library was constructed and sequenced for each triplicate. Sequencing was carried out in a HiSeq 2500 (Illumina) instrument with 100 bases PE reads. Reads were quality trimmed at Q35 with the script prinseq-lite, and only those that exceeded 50 bases in length were kept. Singletons were also excluded from the clean read set. For each library, the number of reads pairs obtained ranged between 21.2 and 24.4 millions, and around 99% passed the quality filter.

Mapping was carried out with the program Bowtiew2 using default settings and the *Streptomyces coelicolor* A3 (2) reference genome (GenBank NC_003888). SAM file was converted to BAM format using samtools, then sorted and indexed. Between 95 and 98% of the cleaned-read set was successfully mapped to the reference genome.

Reads for rRNA origin were detected counting those that mapped to the genome coordinates of the genes 16S, 23S and 5S. In all libraries the proportion of ribosomal reads was below 8%.

For the differential expression analysis, strand specific reads were counted for each CDS feature annotated in the genome reference using HTSEQ v0.6.1p1. A table containing the raw read counts for each CDS feature was generated and used for the subsequent comparative analysis using the BIOCONDUCTOR package EDGER v3. The analysis was performed using the protocol described for RNA-Seq experiments in the user’s guide revised in April 20th 2016.

The RNA-Seq data analyzed here has been deposited in the Sequence Read Archives (SRA, Accession code: PRJNA380047).

## Results

### Aor1 Acts as a Positive Regulator of Antibiotic Production and Differentiation

**FIGURE 1:**
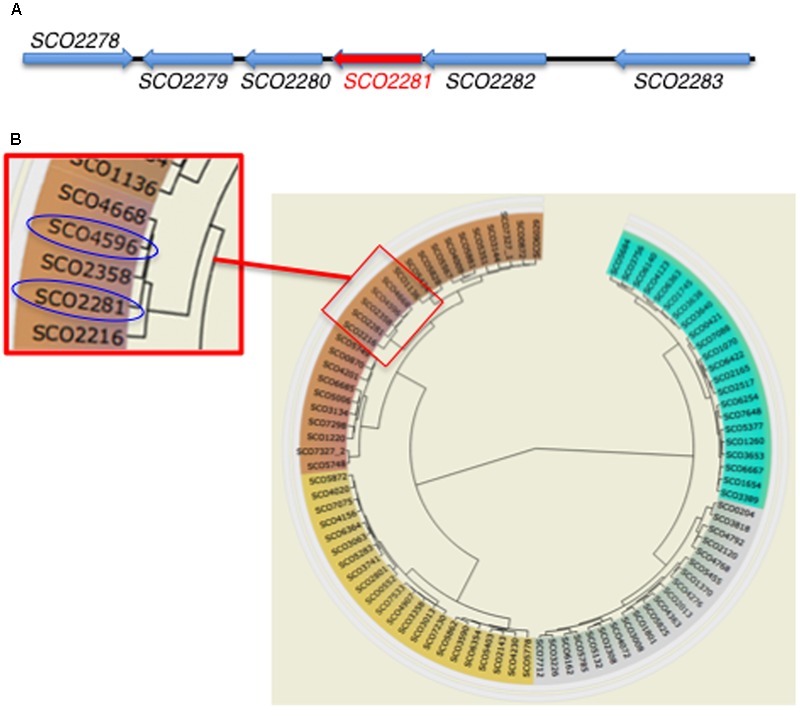
Genomic information of orphan RR *aor1* (*SCO2281*) gene. **(A)** Genomic context (*SCO2278*: Hypothetical protein (HP), *SCO2279*: HP, *SCO2280*: putative TetR regulator, *SCO2282*: HP, *SCO2283*: putative secreted esterase) (source: StrepDB database). **(B)** Circular dendrogram from *S. coelicolor* RRs. Detailed red box: phylogenetic relationship between *SCO2281* and *abrC3* (*SCO4596*) RRs (source: P2CS database).

The gene *aor1* (*SCO2281*) from *S. coelicolor* has 687 nucleotides and 73.36% GC content and encodes a putative NarL orphan RR containing two domains: a REC domain (position 9–128) containing the phosphoaceptor Asp from the HK (CheY-homologous receiver) and an HTH-LuxR DNA binding domain (position 156–218) (SMART database^3^). The encoded protein contains 228 amino acids, with an isoelectric point of 5.47 and a molecular weight of 24.68 kDa. It is highly conserved in all of the *Streptomyces* spp. sequenced to date, with identities ranging from 99 to 75%. Since Aor1 is an orphan RR, there is no HK codifying gene within its genomic context (**Figure 1A**). The phylogenetic circular dendrogram of *S. coelicolor* RRs (P2CS database) shows that *aor1 (SCO2281)* and *abrC3* (*SCO4596*) are closely related and share 59% protein sequence identity (**Figure 1B**). This fact points to the possibility of crosstalk between the HKs AbrC1 and/or AbrC2 of the AbrC system and Aor1. In addition, the other closely related RR gene *SCO2358* shares 55.7% protein sequence identity with Aor1, and forms part of an operon with the HK *SCO2359*. Remarkably, the P2CS database also considered the three genes comprising the AbrC TCS as orphans because the intergenic space between the genes is large enough to contain a promoter region. Moreover, the Prediction of Interaction Specificity in Two-Component Systems website of Biozentrum^4^ showed that there were 20 orphan kinases and 29 orphan regulators in *S. coelicolor*, including the AbrC genes. The existence of a common RNA has been shown for the two HKs AbrC1 and AbrC2, however, no common RNA has been obtained for the HK AbrC2 and the RR AbrC3 (Rodríguez et al., 2015). Using this prediction tool, the HKs AbrC1 (SCO4598) and AbrC2 (SCO4597) were in forth and fifth place as being probable partners with Aor1 (after SCO3750, SCO6424, and SCO0211).

**FIGURE 2:**
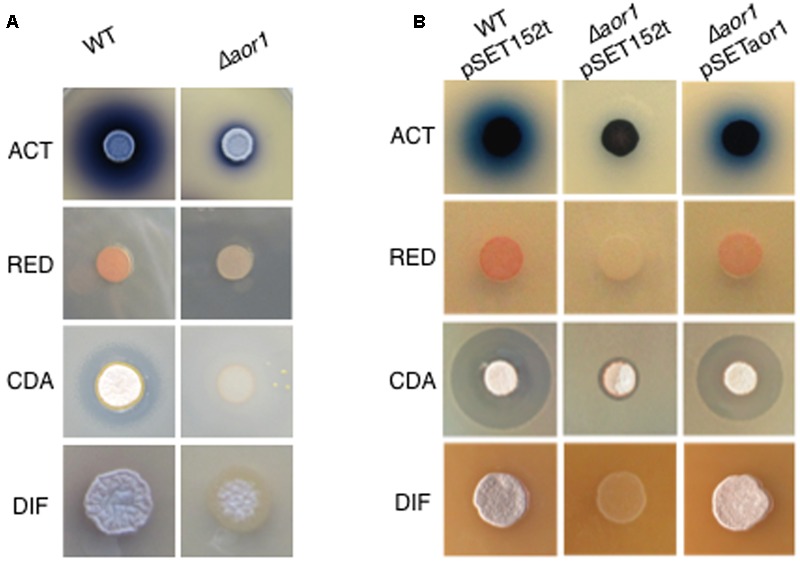
Phenotypes of mutant Δ*aor1.* **(A)** Comparison of phenotypes of Δ*aor1* with wild-type strain. **(B)** Complementation of the Δ*aor1* phenotypes by the integrative plasmid pSETaor1 (pSET152t derivative). ACT, actinorhodin; RED, undecylprodiginine; CDA, calcium dependent antibiotic; DIF, morphological differentiation.

The functional role of Aor1 was analyzed replacing the gene by an apramycin resistance cassette, using the REDIRECT technology (Material and Methods). This mutant strain exhibited a phenotype of an hypo-production of the antibiotics ACT, RED, and CDA, and a delayed morphological development in different media (Supplementary Figure S1), with more extreme phenotypes than those reported for the Δ*abrC* mutant strain (Rico et al., 2014a). Due to the drastic phenotype (**Figure 2A**), and highly reproducibility found in LB medium, complementation assays and RNA-seq analysis (see below) were performed in that medium. These results suggest that Aor1, as previously shown for AbrC3, acts as a positive RR of antibiotic production and differentiation. The Δ*aor1* mutant phenotypes were complemented by the single copy integrated plasmid pSETaor1 (Supplementary Table S1), which discarded the possibility of polarity effects caused by the mutation as the origin of the phenotypes (**Figure 2B**).

### The Gene *aor1* Is Part of a Four-Gene Operon

As shown in **Figure 1A**, *aor1* is located in the vicinity of three genes; two of them (*SCO2279* and *SCO2282*) have been annotated as hypothetical proteins, while *SCO2280* has been annotated as a TetR family transcriptional regulator.

**FIGURE 3:**
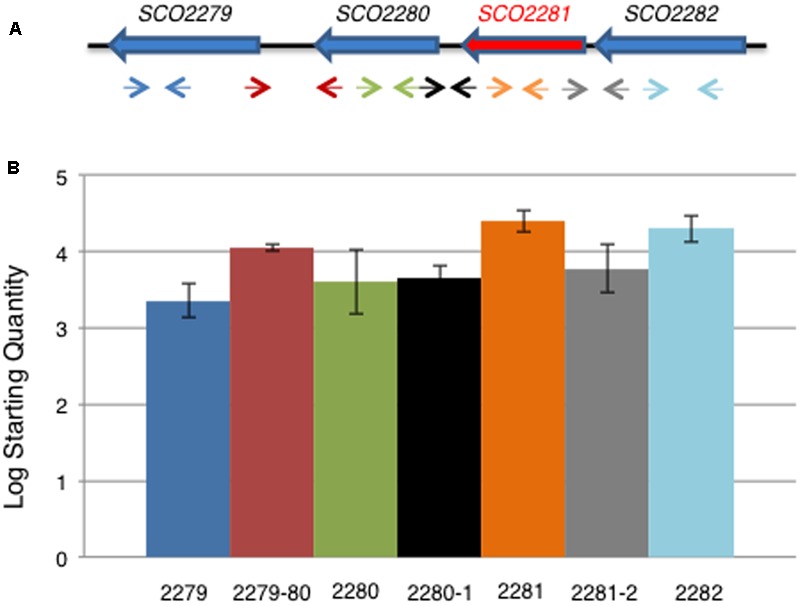
qRT-PCR of *aor1* operon genes. **(A)** Scheme of the gene structure of the putative *aor1* operon and location of the oligonucleotides used. **(B)** Expression profile found by qRT-PCR of the four genes of the *aor1* putative operon and the corresponding intergenic regions between the different genes in NB medium at 48 h. Data represent the Log Starting Quantity of each sample.

In order to determine if all four genes were co-transcribed and participants of a common regulation, qRT-PCR experiments were carried out to detect transcripts within the intergenic regions between the genes. It was found that the intergenic regions were not large enough to contain promoters regions, except for the region between *SCO2279* and *SCO2280* and *SCO2280* and *SCO2281*. qRT-PCR experiments using primers designed to these intergenic regions (Supplementary Table S2) were positive and showed the amplification of a transcript in all cases (**Figure 3**), revealing the existence of a transcript that included all of the genomic region from *SCO2279* to *SCO2282*. This result suggested the existence of an operon with the promoter region located upstream of *SCO2282*.

### Overexpression of *aor1* Does Not Provoke Increased Antibiotic Production

Overexpression of the *aor1* gene was performed under the control of the *SCO2282* upstream region, which corresponded to the promoter region of the *aor1* operon. The plasmid pNaor1 was obtained (Material and Methods) and introduced into the M145 wild-type strain. Antibiotic production was compared to the strain transformed with the empty plasmid used as control on LB agar plates and in liquid LB medium. The high copy number plasmid used to overexpress of the *aor1* gene did not provoke an increase in the production of the antibiotic ACT, as would be expected if it were a positive regulator. This result suggests that Aor1 could possibly be exerting negative control over other negative regulator/s, a situation that will be addressed in the “Discussion” section of this paper (Supplementary Figure S2).

### The Δ*aor1* Mutant Transcriptomic Profile Compared to Wild Type

**FIGURE 4:**
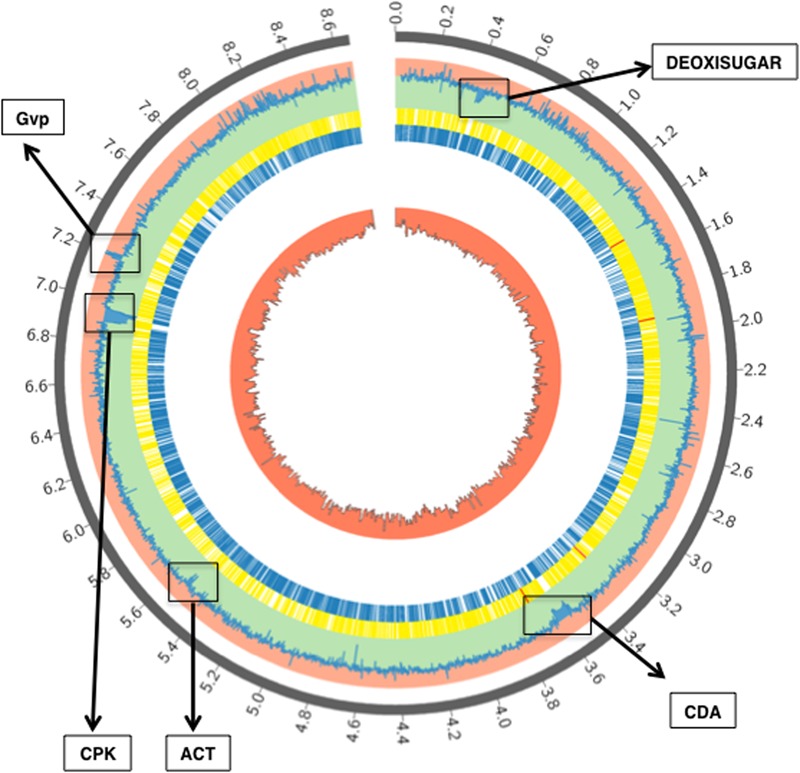
RNA-Seq gene coverage results of *S. coelicolor M145 vs.*Δ*aor1.* Description from the outer circle to the inner one: gray: chromosome scale (Mb); pale blue: logFC M145 vs. Δ*aor1* strain (toward rose zone genes up-regulated in Δ*aor1* and toward green zone genes down-regulated in Δ*aor1; yellow:* CDS in minus strand; blue: CDS in plus strand; red: GC percentage. Some of the most significant clusters differentially expressed are depicted: deoxisugar, CDA, ACT, CPK and Gvp (Gas vesicle proteins).

To study the role of the Aor1 RR in a gene regulatory cascade in *S. coelicolor*, we compared the transcriptome of *S. coelicolor* M145 with *S. coelicolor* Δ*aor1.* Stranded RNA-Seq was performed using RNA from samples of each strain, in triplicate, at 36 h (Material and Methods). The principal component analysis (PCA) and the sample distance matrix showed a distinct gene expression signature between M145 and the Δ*aor1* mutant strains (Supplementary Figures S3, S4). According to the PCA, the first principal component accounted for 82.5% of the variance, and clearly separated both groups. Moreover, the global analysis of the data showed that the absence of the *aor1* gene provoked a huge impact on the transcriptome (**Figure 4** and Supplementary Figures S3, S4).

To facilitate the analysis of the RNA-Seq data, an additional analysis was performed but only on those transcripts whose levels changed at least three-fold in the Δ*aor1* mutant compared with the levels in M145. Five hundred and four genes showed high differential expression (287 down-regulated and 217 up-regulated) between both strains in the conditions studied, with a significant *p*-value ≤ 0.002 and FDR ≤ 0.01. A complete list of the genes corresponding to these transcripts can be found in Supplementary Tables S3, S4. These selected differentially expressed genes were categorized into seven general clusters of functional categories, using David Gene Ontology analysis terms as the reference (Huang da et al., 2009a,b), and are listed in **Table 1**. The relevance of the differentially expressed genes and their correlation with the phenotypic changes observed in the Δ*aor1* mutant were analyzed (see below). Several genes were chosen to perform q-RT-PCR and validate the RNA-Seq results (Supplementary Table S5).

**Table 1:** Differentially expressed genes (RNA-Seq): Functional categories of transcripts whose levels changed at least 3-fold in *S. coelicolor* Δ*aor1* compared with those of M145 (*p*-value ≤ 0.002 and FDR ≤ 0.01).

	Number of genes whose transcripts levels:	
Functional category	Decreased	Increased
*Secondary metabolites (SM)*	93 (32.4%)	3 (1.4%)
Lantibiotic cluster	2	–
Deoxysugar	21	–
Aromatic polyketide	1	–
CDA cluster	33	–
ACT cluster	17	–
Butirolacton	1	–
CPK cluster	16	–
Aromatic polyketide	2	–
Lantibiotic Sap B	–	2
Bacteriocin	–	1
*Regulatory proteins (RP)*	27 (9.4%)	22 (10.1%)
TCSs genes	11	3
Sigma and anti-sigma factors	4	7
Others	12	12
*Gas vesicle proteins (GV)*	1 (0.3%)	12 (5.5%)
*Membrane proteins (MB)*	51 (17.8%)	41 (18.9%)
ABC transporter family	10	6
Other transporter families	10	7
Putative	31	28
*Secreted proteins (S)*	18 (6.3%)	8 (3.7%)
*Others (O)*	30 (10.8%)	61 (28.1%)
*Hypothetical proteins (HP)*	67 (23.3%)	70 (32.7%)
Total	287	217

**FIGURE 5:**
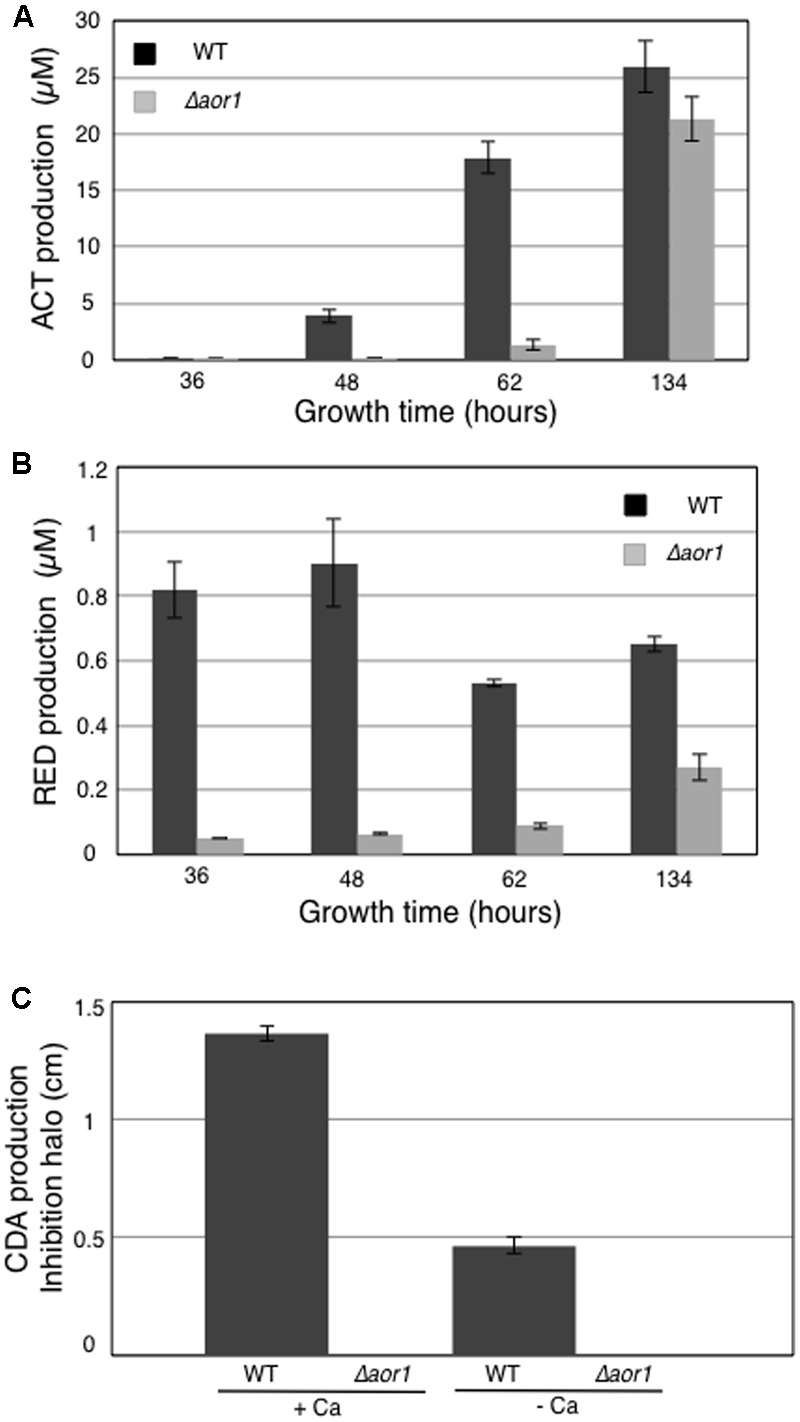
Antibiotic production of *S. coelicolor* M145 (WT) and *S. coelicolor* Δ*aor1* in LB medium. **(A)** Actinorhodin production (ACT). **(B)** Undecylprodiginine production (RED). **(C)** CDA production with and without Ca(NO_3_)_2_ (70 mM) measured in a bioassay against *B. subtilis* using 48 h culture supernatant. Error bar correspond to triplicate measurements.

In addition, samples were collected in parallel at 36, 48, 62, and 134 hours (h) to quantify antibiotic production in the tested growth conditions. ACT production was significantly detected at 48 h in the wild-type strain M145, and this production increased exponentially at 62 and 134 h. However, the Δ*aor1* mutant strain presented very small production even at 62 h, and had a delayed trigger in ACT production at 134 h, yielding slightly lower levels than that of the M145 strain at this time (**Figure 5A**). Regarding RED production, a high level of this antibiotic was produced by M145 at the first time point of 36 h, with a maximum of production at 48 h. RED production in the mutant strain was reduced in all of the times analyzed (**Figure 5B**). Bioassays against *Bacillus subtilis* were used to test the CDA production using the supernatants of 48 h cultures (Material and Methods). There was a small inhibition halo in the M145 strain without calcium that could be due to the production of the other antibiotics (RED and ACT). This halo was much more considerable with plates containing calcium, corresponding to CDA production. In the mutant strain, no inhibition halo was observed in the two conditions used (+Ca and –Ca), indicating that the mutant strain produced less of this antibiotic (**Figure 5C**).

#### Genes Implied in Secondary Metabolism Affected

The expression of 93 out of the 287 down-regulated genes found in the *S. coelicolor* Δ*aor1* mutant strain corresponded to the genes of many clusters encoding secondary metabolites (SM) (32.4%) (Nett et al., 2009). Among these genes, most of the ACT genes clustered together (*SCOs5071-5092;* **Figure 4**, coordinates 5514323–5534546; Supplementary Figure S5) and CDA (*SCOs3210-3249;* **Figure 4**, coordinates 3519449–3602320; Supplementary Figure S5) were found; a result that is in agreement with the phenotype observed in Δ*aor1* strain, including the cluster situated regulators (CSR) genes *actII-ORF4* (*SCO5085*) and *cdaR* (*SCO3217*), although this latter with a FC of 1,9 (Supplementary Table S3). Also, almost all of the genes within several clusters, whose products were not assayed in this study, were found to be down-regulated such as a deoxysugar (*SCOs0381-0401;* **Figure 4**, coordinates 395939–423596), a CPK (*SCOs6273-6288;* **Figure 4**, coordinates 6900898–6907356) and its CSR *cpkO* (*SCO6280*), some genes within the lantibiotic cluster (*SCOs0267-0270*), two aromatic polyketides (*SCO1266* and *SCOs7669-7671*) and the butirolactone (*SCOs6265-66*; **Figure 4**, coordinates 8493549–8496340). Nevertheless, only 3 genes out of 217 (1.4%) in this functional category of secondary metabolites were found to be up-regulated in the Δ*aor1* strain, which were a gene from a bacteriocine cluster (*SCO0753*) and two genes of the lantibiotic cluster containing the SapB gene (*SCOs6682-85;* **Figure 4**, coordinates 7422494–7427004).

#### Genes Encoding Regulatory Proteins Are Affected

Another group of genes with a high percentage of differential expression between the wild-type and the Δ*aor1* mutant strain corresponded to the genes encoding regulatory proteins. As mentioned above, CSRs (SARP proteins coding genes regulating antibiotic production) were down regulated, such as *actII-ORF4, cdaR, cpkO*, and also two CPK regulatory proteins (*SCO6285* and *SCO6288*). This was also the case of ScbR (*SCO6265*), AtrA (*SCO4118*), Aba-like (*SCO4214*), 2 sigma factors including SigU, (*SCO2954*) and 2 putative sigma factors, 6 genes from different regulatory families (LysR, LacI, GntR and, TetR) and 11 TCS genes (including one orphan RR and, two orphan HKs) (**Table 1**). A total of 27 genes of regulatory proteins had a significantly reduced level of expression in the mutant strain (more than 3-fold), corresponding to 19.4% of the down-regulated genes.

On the other hand, 22 genes of this functional category were up-regulated (10.1%), and of these 7 were sigma or anti-sigma factors. These up-regulated genes included the SigB encoding gene (*SCO0600*), its reported regulatory network that is triggered by osmotic or heat shock (SigL, SigM, SigH, SigK), and the anti-anti-SigB RsbV (*SCO7325*) (Supplementary Figure S5 and Supplementary Table S4) (Lee et al., 2004a; Facey et al., 2011), whose relevance in the Δ*aor* mutant will be discussed below.

#### Genes Related to Osmotic/Heat Protection Differentially Expressed

Moreover, in addition to the regulatory genes mentioned above, many other genes regulated by SigB (Lee et al., 2004b) were also over-expressed in the Δ*aor1* mutant compared to the wild-type. These included: *catB* (*SCO0666*) (Cho et al., 2000), coding for a catalase that was 11.4-fold up-regulated; *trxC*, coding for a thioredoxin C (4.7-fold); *ssgC* (*SCO7289*) implied in differentiation (8.4-fold); 12 genes of an operon for gas vesicles proteins (GV) (*SCOs6496-6508;* **Figure 4**, coordinates 7189075-7199188), whose promoter is regulated by SigB and were more than 5-fold up-regulated (Supplementary Table S4); and another 15 genes, some of them with an unknown function.

Other genes not dependent on SigB and related to osmotic protection were also up-regulated, such as a putative osmoprotectant transporter (*SCO1225*), the bacterioferritine gene (*SCO2113*), and *SCO2641* a putative resistance protein implied in detoxification^5^).

#### Membrane and Transport Proteins Differentially Expressed

Those genes that encoded membrane proteins among the differentially expressed genes (92 genes, 51 down- and 41 up-regulated, 17.8 and 18.9 % respectively) were also overrepresented. These genes included 33 members of different superfamilies of transporter proteins (20 down- and 13 up-regulated), such as the ABC (ATP-binding Cassette), MFS (Mayor Facilitator Superfamily), and RND (Resistance-Nodulation-Cell Division) protein superfamilies, among others. Four of the down-regulated putative membrane proteins formed part of the regulon reported for SigU (also down-regulated as mentioned previously), as was the case for 3 putative secreted proteins found to be down-regulated (Gordon et al., 2008). In fact, 24 genes of the secreted proteins were also found to be differentially expressed; 18 genes were down-regulated and 8 up-regulated (6.3 and 3.7 %, respectively).

## Discussion

Due to the increasing number of antibiotic resistant bacteria it is important to revitalize the discovery of new antibiotics (Demain, 2014). In addition, the study of the regulatory networks that control their biosynthesis is also a key goal. In this work, we report the importance of the orphan RR Aor1, which could be considered a positive regulator of antibiotic production and morphological differentiation based on the observed phenotypes of the null mutant (**Figures 2, 5** and Supplementary Figure S1), although part of its action seems to be done through the inactivation of some inhibitors, as discussed below.

The RNA-Seq data have provided a revealing look at the differences between the transcriptomes of *S. coelicolor* M145 and Δ*aor1*, showing that Aor1 is a global regulator that acts in a very high level of the complex regulatory network. In the *S. coelicolor*Δ*aor1* strain, deletion of the *aor1* gene led to significant changes in the global transcriptional landscape, with a three-fold differential expression of 504 genes (287 down-regulated and 217 up-regulated) as compared to the wild-type strain.

In the RNA-Seq data analysis it was found that Aor1 was necessary for triggering the expression of many of the genes encoding the 30 putative secondary metabolites of *S. coelicolor* (Nett et al., 2009). In fact, in addition to the 93 genes down-regulated (32.4%) in Δ*aor1*, as described in the Results section, many other genes in this functional category were down-regulated in the Δ*aor1* mutant, with a significant FDR and *p*-value. However, the differences with respect to the wild-type were less than three-fold (as in the case of *cdaR*). For example, 6 out of 22 genes of the RED cluster (*SCOs5877-5898*) and 4 genes of the eicosapentanoic acid cluster (*SCOs0124-0127*) were found with a FC > 1.7. In contrast, the genes included in the 5-hydroxiectoin cluster (*SCOs1864-1867*) were up-regulated (FC > 1.69) (Supplementary Tables S3, S4). As shown in **Figure 5**, RED production was triggered before 36 h, indicating that the biosynthetic genes were produced before this time. Additionally, this result helps to explain why the differential expression of these genes observed at 36 h was not as significant as the one observed for ACT or CDA.

The onset of some of these secondary metabolites by Aor1 seems to be mediated by the activation of other regulatory proteins that have been previously reported to control antibiotic production (all of them down regulated in the Δ*aor1*) such as the antibiotic CSRs, ActII-ORF4, CdaR and, CpkO, the γ-butirolactone (ScbA) and its regulator (ScbR), AbaA-like and, AtrA (Liu et al., 2013). Other regulatory genes were also down- or up-regulated, including global regulators like TCSs that have not been characterized yet. It is also worth mentioning that in the CDA cluster (*SCOs3210-3279*), only the RR gene AbsA2 (*SCO3226*) was up-regulated (with a FC = 1.8) (Supplementary Table S4). Therefore, the regulation of some of the CSRs by Aor1 might be partly due to a negative regulation of AbsA2, which has been reported to be a negative global regulator (Sheeler et al., 2005). This could explain why deletion of the *aor1* gene had such strong phenotypic effects and that a drastic increment in antibiotic production was not observed with a high copy number plasmid expression harboring *aor1*.

On the other hand, the regulatory network and targets that respond to osmotic stress and heat shock mediated by the master sigma factor SigB, such as SigL, SigM, SigH, SigK, CatB, TrxC, SsgC and a whole gas vesicle (GV) operon (Lee et al., 2004b, 2005; Facey et al., 2011), were found up-regulated in the *S. coelicolor* Δ*aor1* mutant strain. The genes encoding WhiB (*SCO3034*) (indirect target of SigB) and DpsA (*SCO0596*) (direct target of SigH and SigB) were also differentially expressed and were up-regulated by 2.68-fold and 2.6-fold, respectively.

**FIGURE 6:**
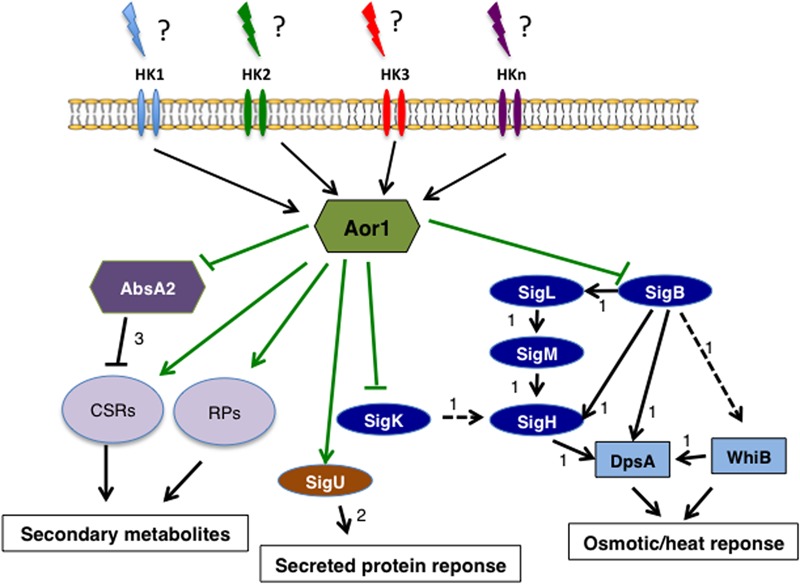
Scheme of the Aor1 network of *S. coelicolor.* The schematic model shows the putative gene network governed by Aor1 and its pleiotropic regulatory roles that connect secondary metabolism, secreted protein response mediated by SigU, and osmotic/heat response mediated by Sig B. Arrow: positive control; blunt line: negative control; solid black arrow: direct control; dashed black arrow: indirect control. Green lines: this work (to determine the direct or indirect control). The data for arrows 1 are from the work of Facey et al. (2011); arrow 2 from Gordon et al. (2008); CSR, Cluster Situated Regulators; RPs, Regulatory proteins.

One hypothesis, that needs to be addressed in the future, is if Aor1 may be modulating the osmotic stress response and antibiotic production of the cell, integrating different external signals through HKs (**Figure 6**). The *aor1* deletion triggered the cellular responses mediated by SigB regulon in order to protect the cell against osmotic stress as reported for the GV operon (van Keulen et al., 2005), trxC and catB. Thus, Aor1 could be acting as a negative regulator of *sigB*. Several osmotic stress related responses, not yet described as being dependent on SigB, have also been found to be up-regulated, such as the already mentioned putative osmoprotectant transporter of proline/betaine from the MFS (*SCO1225*)^6^ and 5-hydroxiectoin compatible solute’ overexpression (with a FC > 1.7) (Eilert et al., 2013; Sadeghi et al., 2014a,b). At the same time, a general decrease in secondary metabolite production in the Δ*aor1* mutant could take place, as explained above. These results are in accordance with the inverse correlation between osmotic stress response and antibiotic production reported previously in *Streptomyces avermitilis* (Godinez et al., 2015). A precocious overproduction of ACT has also been reported in several *S. coelicolor sigB* mutant strains, reinforcing this correlation (Cho et al., 2001; Fernández Martínez et al., 2009). Moreover, as part of the stress response provoked by the absence of Aor1, the transport functions of the mutant cells seemed to be altered, owing to the high number of transporter proteins that were found affected, as the mentioned above *SCO1225.* In addition, when mutant cells sense osmotic stress they need to control their secretion in order to maintain high intracellular osmolarity. Similarly, it has also been reported that the extracytoplasmic function sigma factor (ECF) SigU elicits secretion and increases protease activity. In the Δ*aor1* mutant, the secreted and membrane proteins whose expression is triggered by SigU are down-regulated, and the protease inhibitor Sti1 (*SCO0762*) is 2.5-fold up-regulated. These results are in line with this working model (Supplementary Table S4) (Gordon et al., 2008).

Since Aor1 is an orphan RR, this opens up the possibility for it to be phosphorylated by different HKs, (Whitworth and Cock, 2009) allowing Aor1 to respond to many environmental cues that might exert a strict control on the osmotic stress response mediated by SigB and correct secondary metabolite production. Clarifying the function of Aor1 and situating it in a concrete point of the *S. coelicolor* complex regulatory network is a difficult task, as is demonstrated by the few regulators with known stimuli found in *Streptomyces* (Rodríguez et al., 2013). This task acquires an additional difficulty level in the case of orphan regulators. Relation between an orphan regulator and an HK has rarely been demonstrated previously, but combination of BLASTP search and trans-phosphorylation may serve to elucidate the role of Aor1 in the future (Wang et al., 2009). Several HKs have been reported as probable partners of Aor1 such as the above-mentioned AbrC1, AbrC2 (Rodríguez et al., 2015), SCO3750, SCO6424, and SCO0211. The phenotypes observed in Δ*aor1* were more drastic than those reported for Δ*abrC*, reinforcing the idea that more than one HK might be controlling the activity of this orphan regulator. As mentioned before, *Streptomyces* has a high number of TCSs due to its complex lifestyle and genome size, and several TCSs have been found down-regulated in Δ*aor1*, which also suggests a transcriptional cross-regulation exerted by Aor1 on these signal transduction systems. As Zhou et al. stated, key features of *Streptomyces*, such as gene regulation, stress response, secondary metabolism, and morphological differentiation, are not stand-alone properties but are related to each other (Zhou et al., 2011). Aor1 may be establishing a connection that integrates some of these pathways in a high level of the regulatory network. Other TCSs as OsdK/R (SCO0203/0204), (Urem et al., 2016) and OsaA/B (SCOs5748/5749) have also been reported to respond to oxygen and osmotic stress responses, respectively (Godinez et al., 2015).

## Conclusion

It has been demonstrated using transcriptome profiling that the *aor1* deletion results in a highly altered transcription pattern compared to that of the parental strain. Thus, these results highlight that Aor1 plays a role in the regulation of genes related to osmotic stress and secondary metabolite production in *S. coelicolor*. Nevertheless, these data include changes that are both direct and indirect results of the effects of the orphan RR Aor1 on gene expression. Further experiments, such as a ChIP-Seq, must be carried out in the future to determine the direct targets of this RR. Another challenge is finding the corresponding cognate HKs, which to date has proven to be a difficult task.

## Author Contributions

SA, SR, HR, LS, and RS conducted the experiment(s). SA, JA, and MD analyzed the results. SA, RS, and MD conceived the experiment(s) and wrote the manuscript. All authors read and approved the final manuscript.

## Conflict of Interest Statement

The authors declare that the research was conducted in the absence of any commercial or financial relationships that could be construed as a potential conflict of interest.

## Footnotes

1 http://www.p2cs.org/

2 https://sourceforge.net/projects/primer3/

3 http://smart.embl-heidelberg.de/

4 http://www.swissregulon.unibas.ch/cgi-bin/TCS.pl

5 http://dbkgroup.org/s_coeli/referencegel/proteinDetails.php?proteinId=SCO2641

6 http://www.membranetransport.org/other_type.php?fType=Secondary+Transporter&oOID=scoe1
