# Supplementary material for: The Orphan Response Regulator Aor1 Is a New Relevant Piece in the Complex Puzzle of Streptomyces coelicolor Antibiotic Regulatory Network
Source: Front Microbiol. 2017 Dec 12;8:2444. doi: 10.3389/fmicb.2017.02444 (PMC5733086; doi:10.3389/fmicb.2017.02444)
Supplement: Supplementary file 1 [file Table_1.pdf]

Supplementary Table S1: *Plasmids and cosmids used in this work*

Vector	Characteristics	Reference
pIJ790	λ-RED (*gam. beta. exo*). *cat. araC. rep101ts*	[1]
pIJ773	*aac(3)IV* (ApraR)+*oriT*. FRT sites	[1]
pUZ8002	*tra. neo*. RP4	[2]
SCC30	Supercos-1 derivative: *bla. neo*. Contains gene *aor1*	[3]
ΔSCC30-1	SCC30 *ΔSCO2281:aac(3)IV*	This work
pSET152t	Integrative plasmid with apramycin/tiostreptone resistance	This work
pSETaor1	pSET152t derivative containing *aor1*	This work
pXHis1	E. coli plasmid containing *xysA* gene under *xysA* promoter	[4]
pXHisaor1	pXHis1 plasmid derivative. Contains *aor1* gene under xysA promoter	This work
pHisaor1	pXHis1 plasmid derivative. Contains *aor1* gene under its promoter	This work
pN702GEM3	Bifunctional plasmid *E. coli/Streptomyces* neo	[5]
pNX24	pN702GEM3 plasmid derivative. Contains *xysA* gene under *xysA* promoter	[4]
pNXaor1	pN702GEM3 plasmid derivative. Contains *aor1* gene under *xysA* promoter	This work
pNaor1	pN702GEM3 plasmid derivative. Contains *aor1* gene under its promoter	This work

References

1 Gust. B.. Challis. G. L.. Fowler. K.. Kieser. T. & Chater. K. F. PCR-targeted *Streptomyces* gene replacement identifies a protein domain needed for biosynthesis of the sesquiterpene soil odor geosmin. *Proc Natl Acad Sci U S A* **100**. 1541-1546 (2003).

2 Paget. M. S.. Leibovitz. E. & Buttner. M. J. A putative two-component signal transduction system regulates *sigmaE*. a sigma factor required for normal cell wall integrity in *Streptomyces coelicolor A3*(2). *Mol Microbiol* **33**. 97-107 (1999).

3 Redenbach. M. *et al.* A set of ordered cosmids and a detailed genetic and physical map for the 8 Mb *Streptomyces coelicolor* A3(2) chromosome. *Molecular Microbiology* **21**. 77-96 (1996).

4 Adham. A.I.A. et al. Expression of the genes coding for the xylanase Xys1 and the cellulase Cel1 from the straw-decomposing *Streptomyces halstedii* JM8 cloned into the amino-acid producer *Brevibacterium lactofermentum* ATCC13869. Arch Microbiol 177:91-97 (2001)

5 Fernández-Abalos. J. M. *et al.* Posttranslational processing of the xylanase Xys1L from *Streptomyces halstedii* JM8 is carried out by secreted serine proteases. *Microbiology* **149**. 1623-1632 (2003)
