# Supplementary material for: The Orphan Response Regulator Aor1 Is a New Relevant Piece in the Complex Puzzle of Streptomyces coelicolor Antibiotic Regulatory Network
Source: Front Microbiol. 2017 Dec 12;8:2444. doi: 10.3389/fmicb.2017.02444 (PMC5733086; doi:10.3389/fmicb.2017.02444)
Supplement: Supplementary file 2 [file Table_2.PDF]

Supplementary Table S2: *Oligonucleotides used in this work*

Name	Sequence 5'-3'	Use
SRG-28	GGCGACCCCTGGCCGCCCGGGGAGGACTGA GGAAGAAGTGATTCCGGGGATCCGTCGACC	Forward oligonucleotide to obtain the mutagenic cassette.
SRG-30	ACGGAACGTCTCGCGCCGGCCGTGGGCGGC GCTCCGCTCATGTAGGCTGGAGCTGCTTC	Reverse oligonucleotide to obtain the mutagenic cassette.
SRG-03	TTTTTTCATATGGAACGTGACGTGCGGCCC	Forward oligonucleotide for cloning *aor1*. The sequence recognized by NdeI is underlined.
SRG-04	TTTTTTCTCGAGGTGGCGTGGCGCCCGCGCG	Reverse oligonucleotide for cloning *aor1*. The sequence recognized by XhoI is underlined.
SRG-37	TTTTTTGAATTCGTCCGCGGGGGCGGGTGC	Forward oligonucleotide to amplify *aor1p*. The sequence recognized by EcoRI is underlined.
SRG-38	TTTTTTCATATGCCGGAGATTGTACGAGGCACTC C	Reverse oligonucleotide to amplify *aor1p*. The sequence recognized by NdeI is underlined.
2279 f bis	ATGCTGCTGTTCACCCTCAC	Forward oligonucleotide for q-RT-PCR of *SCO2279*.
2279 r bis	GCGGTACCAGCACATAGGAA	Reverse oligonucleotide for q-RT-PCR of *SCO2279*.
2279-2280 interg f	GTTCTCCCACGCCTACACC	Forward oligonucleotide for q-RT-PCR of *SCO2279-SCO2280* intergenic region.
2279-2280 interg r	GAACTCCGCGCTCATCTATC	Reverse oligonucleotide for q-RT-PCR of *SCO2279-SCO2280* intergenic region.
2280 F	CGTACACCATCCCGGACAC	Forward oligonucleotide for q-RT-PCR of *SCO2280*.
2280 R	GTCGATGTCCTGCCGTACC	Reverse oligonucleotide for q-RT-PCR of *SCO2280*.
2281 f bis	GTCTGGGGTACCTGCTCAAG	Forward oligonucleotide for q-RT-PCR of *SCO2281*.
2281 r bis	GAACAACTGGGCGACGAC	Reverse oligonucleotide for q-RT-PCR of *SCO2281*.
2281-2280 interg f	CGTCCTGCACTACCTGGAGA	Forward oligonucleotide for q-RT-PCR of *SCO2280-SCO2281* intergenic region.
2281-2280	GGTTCGTGATGCGTTTCC	Reverse oligonucleotide for q-RT-PCR of

interg r		*SCO2280-SCO2281* intergenic region.
2281-2 interg f bis	GCGGAGTACGGTCACCAAG	Forward oligonucleotide for q-RT-PCR of *SCO2281-SCO2282* intergenic region.
2281-2 interg r bis	CGTCACGTTCCACTTCTTCC	Reverse oligonucleotide for q-RT-PCR of *SCO2281-SCO2282* intergenic region.
2282 f bis	GCGTACGAGGACGAGAAGT	Forward oligonucleotide for q-RT-PCR of *SCO2282.*
2282 r bis	CTCGTCGGATGCACACAG	Reverse oligonucleotide for q-RT-PCR of *SCO2282.*
CDA-F	CTGAGAACTCTTCGGTTCGG	Forward oligonucleotide for q-RT-PCR of *SCO3230.*
CDA-R	CAGGATCCCGTCCGGTAG	Reverse oligonucleotide for q-RT-PCR of *SCO3230.*
SigH-F	CCCTGGACGACCTGACC	Forward oligonucleotide for q-RT-PCR of *SCO5243.*
SigH-R	GGAAGTGCCGCTTGATCTC	Reverse oligonucleotide for q-RT-PCR of *SCO5243.*
DpsA-F	AGCGGAAGTGGGACGACTAC	Forward oligonucleotide for q-RT-PCR of *SCO0596.*
DpsA-R	TCAGAAGGTCCTCGGTGGC	Reverse oligonucleotide for q-RT-PCR of *SCO0596.*
ACT-F	AAAGCAATATCGCGCACCTGGAAG	Forward oligonucleotide for q-RT-PCR of *SCO5085.*
ACT-R	GTTCCGGAATCATCGGCCCTATTC	Reverse oligonucleotide for q-RT-PCR of *SCO5085.*
CPK-F	TCAGTGTGCTCCTCGTCCAGAAC	Forward oligonucleotide for q-RT-PCR of *SCO6280.*
CPK-R	TTGAGGTGCTCGAAGTCCTCGTC	Reverse oligonucleotide for q-RT-PCR of *SCO6280.*
GVP-F	GATCTCTGAGGTCCTGCTGC	Forward oligonucleotide for q-RT-PCR of *SCO6502.*
GVP-R	TGGCCGGGTCGTAGTAGATC	Reverse oligonucleotide for q-RT-PCR of *SCO6502.*
3603-F	CATCATCGCCGCCTACCTC	Forward oligonucleotide for q-RT-PCR of *SCO3603.*
3603-R	CATGACGAACAGCCCGATCT	Reverse oligonucleotide for q-RT-PCR of *SCO3603.*
