# Supplementary material for: The Orphan Response Regulator Aor1 Is a New Relevant Piece in the Complex Puzzle of Streptomyces coelicolor Antibiotic Regulatory Network
Source: Front Microbiol. 2017 Dec 12;8:2444. doi: 10.3389/fmicb.2017.02444 (PMC5733086; doi:10.3389/fmicb.2017.02444)
Supplement: Supplementary file 3 [file Table_3.PDF]

Supplementary Table S3: *Genes differentially down-regulated in ∆aor1 (RNA-Seq)*

Genes whose transcript levels changed at least 3-fold (FC) *in S. coelicolor* ∆*aor1* compared with those of M145. p-value ≤ 0.002 and FDR ≤ 001 (* FC: relevant genes with FC<3). Secondary metabolites: SM; Regulatory proteins: RP; Membrane proteins: MB; Gas vesicle proteins: GV; Hypothetical proteins: HP; Secreted proteins: S; Other functions: O.

Gene	FC	p- value	FDR	Function	Regulon [1]
SCO0039	3.22E+00	7.38E-07	6.52E-06	hypothetical protein (HP)	
SCO0070	3.16E+00	2.29E-05	1.24E-04	hypothetical protein (HP)	
*SCO0124**	1.72E+00	3.18E-03	7.91E-03	eicosapentaenoic acid cluster (SM)	
*SCO0125**	2.12E+00	3.10E-04	1.11E-03	eicosapentaenoic acid cluster (SM)	
*SCO0126**	1.98E+00	1.27E-05	7.54E-05	eicosapentaenoic acid cluster (SM)	
*SCO0127**	2.29E+00	2.24E-08	2.85E-07	eicosapentaenoic acid cluster (SM)	
SCO0224	1.34E+01	5.79E-09	8.36E-08	conserved hypothetical protein (HP)	
SCO0269	3.20E+00	2.97E-06	2.17E-05	lantibiotic cluster (SM)	
SCO0270	3.07E+00	3.93E-07	3.74E-06	lantibiotic cluster (SM)	
SCO0320	2.37E+01	1.23E-12	2.81E-11	hypothetical protein (HP)	
SCO0321	3.74E+00	1.81E-08	2.38E-07	putative carboxylesterase (O)	
SCO0381	2.12E+01	4.36E-32	3.32E-30	deoxysugar cluster (SM)	
SCO0382	1.68E+01	9.15E-27	5.05E-25	deoxysugar cluster (SM)	
SCO0383	1.41E+01	2.10E-21	8.63E-20	deoxysugar cluster (SM)	
SCO0384	1.54E+01	1.77E-24	8.31E-23	deoxysugar cluster (SM)	
SCO0385	1.52E+01	2.58E-25	1.32E-23	deoxysugar cluster (SM)	
SCO0386	1.37E+01	6.66E-32	5.03E-30	deoxysugar cluster (SM)	
SCO0387	1.37E+01	1.10E-30	7.64E-29	deoxysugar cluster (SM)	
SCO0388	1.57E+01	1.16E-28	7.12E-27	deoxysugar cluster (SM)	
SCO0389	1.17E+01	1.50E-33	1.22E-31	deoxysugar cluster (SM)	

SCO0390	1.09E+01	1.87E-26	1.02E-24	deoxysugar cluster (SM)	
SCO0391	1.88E+01	1.90E-42	2.23E-40	deoxysugar cluster (SM)	
SCO0392	1.52E+01	1.10E-36	1.04E-34	deoxysugar cluster (SM)	
SCO0393	1.39E+01	1.90E-32	1.46E-30	deoxysugar cluster (SM)	
SCO0394	1.23E+01	4.94E-37	4.74E-35	deoxysugar cluster (SM)	
SCO0395	1.11E+01	1.25E-28	7.61E-27	deoxysugar cluster (SM)	
SCO0396	1.02E+01	2.97E-31	2.13E-29	deoxysugar cluster (SM)	
SCO0397	1.11E+01	1.12E-29	7.31E-28	deoxysugar cluster (SM)	
SCO0398	6.80E+00	1.92E-20	7.58E-19	deoxysugar cluster (SM)	
SCO0399	9.83E+00	1.08E-25	5.68E-24	deoxysugar cluster (SM)	
SCO0400	7.75E+00	6.46E-30	4.26E-28	deoxysugar cluster (SM)	
SCO0401	9.66E+00	1.90E-24	8.87E-23	deoxysugar cluster (SM)	
SCO0409	5.64E+00	1.35E-29	8.74E-28	spore asociated protein (O)	
SCO0532	3.08E+00	1.57E-06	1.25E-05	putative sugar transporter membrane protein ABC (MB)	
SCO0548	3.37E+00	9.55E-05	4.18E-04	putative 3-oxoacyl-(acyl-carrier-protein) synthase (O)	
SCO0554	3.15E+00	2.23E-07	2.28E-06	putative secreted beta-mannosidase (S)	
SCO0644	4.13E+00	1.81E-14	4.70E-13	putative membrane protein MB)	SigU
SCO0656	3.39E+00	1.12E-07	1.22E-06	gas vesicle (GV)	
SCO0685	3.28E+00	5.09E-07	4.71E-06	hypothetical protein (HP)	
SCO0729	3.28E+00	2.67E-19	9.57E-18	conserved hypothetical protein (HP)	
SCO0752	4.47E+00	2.28E-22	9.62E-21	secreted serin protease (SalO) (S)	SigU
SCO0882	3.35E+00	3.70E-06	2.61E-05	hypothetical protein (HP)	
SCO0909	3.62E+00	2.05E-20	8.02E-19	conserved hypothetical protein (HP)	
SCO0930	5.49E+00	3.81E-21	1.55E-19	putative lipoprotein (MB)	SigU
SCO0944	4.77E+00	7.39E-29	4.59E-27	putative membrane protein (MB)	
SCO0973	9.18E+00	1.24E-23	5.58E-22	putative integral membrane protein (MB)	
SCO0991	3.34E+00	1.14E-08	1.57E-07	conserved hypothetical protein (HP)	

SCO1053	3.24E+00	9.66E-06	5.94E-05	hypothetical protein (HP)	
SCO1063	3.67E+00	5.79E-08	6.72E-07	putative sugar transport integral membrane protein ABC (MB)	
SCO1065	3.67E+00	2.73E-12	6.00E-11	putative sugar transport sugar binding protein ABC (T)	
SCO1072	3.33E+00	1.06E-06	8.84E-06	hypothetical protein (HP)	
SCO1100	3.20E+00	7.43E-12	1.57E-10	putative integral membrane protein (MB)	
SCO1160	3.03E+00	5.89E-06	3.86E-05	putative membrane protein (MB)	
SCO1178	2.21E+01	1.09E-15	3.08E-14	hypothetical protein (HP)	
SCO1227	3.35E+00	5.05E-09	7.38E-08	putative DNA-binding protein (RP)	
SCO1266	3.11E+00	1.33E-05	7.82E-05	aromatic polyketide (SM)	
SCO1281	3.05E+00	2.44E-05	1.32E-04	putative oxidoreductase (O)	
SCO1356	3.62E+00	5.92E-21	2.38E-19	secreted iron sulphur protein (S)	SigU
SCO1357	3.45E+00	8.46E-11	1.60E-09	hypothetical protein (HP)	
SCO1358	3.07E+00	3.54E-09	5.37E-08	LysR-family transcriptional regulator (RP)	
SCO1364	5.81E+00	3.12E-19	1.10E-17	hypothetical protein (HP)	
SCO1366	3.38E+00	1.55E-20	6.17E-19	conserved hypothetical protein (HP)	
SCO1367	2.56E+01	4.32E-65	1.68E-62	ABC transport ATP binding (MB)	
SCO1368	1.69E+01	4.11E-63	1.38E-60	ABC transport (MB)	
SCO1369	5.85E+00	1.33E-30	9.08E-29	two-component system sensor kinase HK (RP)	
SCO1370	4.58E+00	4.90E-33	3.85E-31	response regulator. NarL family (RP)	
SCO1456	3.40E+00	3.00E-05	1.56E-04	hypothetical protein (HP)	
SCO1550	8.48E+00	7.27E-17	2.27E-15	putative small membrane protein (MB)	
SCO1573	4.37E+00	2.72E-22	1.14E-20	putative oxidoreductase membrane protein (MB)	SigU
SCO1574	3.27E+00	4.39E-14	1.11E-12	hypothetical protein (HP)	
SCO1575	3.00E+00	1.45E-10	2.63E-09	putative thiamine biosynthesis lipoprotein precursor (O)	
SCO1722	3.17E+00	3.36E-06	2.41E-05	putative membrane protein (MB)	
SCO1723	3.40E+00	3.69E-06	2.61E-05	RNA polymerase sigma factor (RP)	
SCO1740	3.37E+00	5.21E-05	2.49E-04	putative membrane protein (MB)	

SCO1767	3.87E+00	1.43E-14	3.77E-13	putative DNA hydrolase (O)	
SCO1801	4.59E+00	4.59E-11	8.86E-10	response regulator. NarL family (RP)	
SCO1802	4.14E+00	1.09E-11	2.25E-10	two-component system sensor kinase HK (RP)	
SCO1803	5.45E+01	5.50E-08	6.40E-07	oxido reductase (O)	
SCO1804	4.41E+01	2.50E-08	3.13E-07	SAM tRNA ribosyl transferase (O)	
SCO1839	4.07E+00	5.15E-11	9.89E-10	putative transcriptional regulator (RP)	
SCO1862	5.21E+00	2.06E-18	7.06E-17	putative integral membrane protein (MB)	
SCO1868	4.31E+00	8.54E-21	3.41E-19	conserved hypothetical protein (HP)	
SCO1892	3.12E+00	2.10E-12	4.68E-11	putative integral membrane efflux protein (MSF-MDR) (MD)	
SCO1905	3.31E+00	1.98E-14	5.07E-13	hypothetical protein (HP)	
SCO1909	1.31E+01	3.37E-12	7.38E-11	hypothetical protein (HP)	
SCO2028	3.65E+00	2.59E-05	1.39E-04	putative membrane protein (MB)	
SCO2029	3.03E+00	8.54E-07	7.33E-06	putative secreted protein (S)	
SCO2056	3.04E+00	2.35E-05	1.27E-04	hypothetical protein (HP)	
SCO2217	4.49E+00	8.64E-17	2.64E-15	putative secreted protein (S)	SigU
SCO2218	7.64E+00	3.02E-11	5.96E-10	putative lipoprotein (MB)	
SCO2309	5.76E+00	1.97E-08	2.55E-07	putative transmembrane transport protein MDR (MB)	
SCO2408	3.89E+00	1.65E-14	4.31E-13	putative aminotransferase (O)	SigU
SCO2477	3.15E+00	1.62E-07	1.71E-06	putative 3-oxoacyl-[acyl-carrier protein] reductase (O)	
SCO2478	1.28E+01	8.43E-13	1.95E-11	putative reductase (O)	
SCO2492	8.69E+00	1.44E-25	7.55E-24	putative membrane protein (MB)	
SCO2495	3.43E+00	2.98E-16	8.77E-15	putative membrane protein (MB)	SigU
SCO2511	3.47E+00	3.61E-09	5.44E-08	hypothetical protein (HP)	
SCO2512	5.14E+00	2.08E-17	6.68E-16	hypothetical protein (HP)	
SCO2517	6.86E+00	4.44E-41	4.76E-39	response regulator. NarL family (RP)	
SCO2518	7.99E+00	5.99E-50	7.91E-48	two-component system sensor kinase HK (RP)	
SCO2519	2.36E+01	2.32E-36	2.15E-34	put prot mb MmpL homolog (RND superfamily) (MB)	

SCO2550	3.71E+00	2.61E-17	8.31E-16	putative lipoprotein (MB)	
SCO2641	3.31E+00	8.68E-13	2.00E-11	putative resistance protein (O)	
SCO2695	1.45E+01	7.80E-13	1.83E-11	hypothetical protein (HP)	
SCO2696	4.63E+00	3.25E-08	3.98E-07	putative 2-hydroxyacid-family dehydrogenase (O)	
SCO2706	3.05E+00	2.03E-07	2.10E-06	putative transferase (O)	
SCO2745	3.12E+00	1.30E-12	2.95E-11	probable LacI-family transcriptional regulator (RP)	
SCO2861	3.03E+00	2.16E-08	2.76E-07	hypothetical protein (HP)	
SCO2909	3.11E+00	4.41E-13	1.05E-11	putative membrane protein (MB)	
SCO2937	4.51E+00	1.09E-07	1.19E-06	putative transmembrane transport protein (MB)	
SCO2954	3.00E+00	5.12E-18	1.72E-16	RNA polymerase sigma-70 factor SigU (RP)	SigU
SCO2976	7.47E+00	6.16E-14	1.54E-12	hypothetical protein (HP)	
SCO2977	3.02E+00	1.55E-18	5.34E-17	hypothetical protein (HP)	
SCO3111	3.39E+00	2.92E-11	5.79E-10	putative ABC transport system ATP-binding protein (MB)	
SCO3134	4.18E+00	7.41E-19	2.58E-17	response regulator. NarL family (RP)	
SCO3210	5.91E+00	1.20E-24	5.70E-23	CDA cluster (SM)	
SCO3211	7.17E+00	2.39E-25	1.24E-23	CDA cluster (SM)	
SCO3212	5.72E+00	2.75E-27	1.57E-25	CDA cluster (SM)	
SCO3213	4.54E+00	1.58E-08	2.09E-07	CDA cluster (SM)	
SCO3214	4.86E+00	5.93E-23	2.58E-21	CDA cluster (SM)	
SCO3215	6.70E+00	3.16E-34	2.71E-32	CDA cluster (SM)	
*SCO3217**	1.91E+00	1.71E-04	6.78E-04	CDA cluster (SM). cdaR (RP)	
SCO3218	1.40E+01	2.52E-12	5.57E-11	CDA cluster (SM)	
SCO3220	2.21E+01	1.56E-15	4.31E-14	CDA cluster (SM)	
SCO3221	1.48E+01	2.26E-14	5.79E-13	CDA cluster (SM)	
SCO3222	1.15E+01	4.78E-07	4.45E-06	CDA cluster (SM)	
SCO3227	7.39E+00	1.95E-14	5.03E-13	CDA cluster (SM)	
SCO3228	1.10E+01	2.36E-26	1.26E-24	CDA cluster (SM)	

SCO3229	1.29E+01	6.80E-12	1.45E-10	CDA cluster (SM)
SCO3230	8.81E+00	2.96E-22	1.23E-20	CDA cluster (SM)
SCO3231	7.66E+00	5.49E-25	2.67E-23	CDA cluster (SM)
SCO3232	8.29E+00	3.71E-23	1.63E-21	CDA cluster (SM)
SCO3233	7.41E+00	1.07E-18	3.69E-17	CDA cluster (SM)
SCO3234	7.46E+00	7.35E-17	2.27E-15	CDA cluster (SM)
SCO3235	8.72E+00	2.02E-16	6.08E-15	CDA cluster (SM)
SCO3236	1.27E+01	5.24E-12	1.13E-10	CDA cluster (SM)
SCO3237	6.77E+00	1.01E-14	2.68E-13	CDA cluster (SM)
SCO3238	8.02E+00	2.15E-20	8.38E-19	CDA cluster (SM)
SCO3239	8.53E+00	2.58E-20	9.93E-19	CDA cluster (SM)
SCO3240	1.19E+01	2.33E-38	2.36E-36	CDA cluster (SM)
SCO3241	9.64E+00	3.16E-20	1.20E-18	CDA cluster (SM)
SCO3242	1.80E+01	2.98E-13	7.17E-12	CDA cluster (SM)
SCO3243	1.40E+01	9.49E-16	2.70E-14	CDA cluster (SM)
SCO3244	1.21E+01	2.68E-11	5.33E-10	CDA cluster (SM)
SCO3245	1.16E+01	2.65E-24	1.23E-22	CDA cluster (SM)
SCO3246	9.72E+00	5.86E-11	1.12E-09	CDA cluster (SM)
SCO3247	1.12E+01	2.90E-16	8.57E-15	CDA cluster (SM)
SCO3248	1.32E+01	1.53E-10	2.76E-09	CDA cluster (SM)
SCO3249	1.59E+01	6.31E-16	1.81E-14	CDA cluster (SM)
SCO3263	5.02E+00	9.79E-08	1.08E-06	conserved hypothetical protein (HP)
SCO3264	4.68E+00	1.39E-12	3.14E-11	putative GntR transcriptional regulator (RP)
SCO3287	3.23E+00	3.65E-07	3.49E-06	putative serine/arginine rich protein (O)
SCO3713	3.44E+00	7.08E-08	8.04E-07	hypothetical protein (HP)
SCO3714	4.87E+00	1.29E-10	2.37E-09	putative transposase (O)
SCO3750	3.78E+00	7.98E-20	2.96E-18	two-component system sensor kinase HK (RP)

SCO3999	3.71E+00	9.97E-20	3.67E-18	putative lipoprotein (MB)
SCO4005	5.24E+00	1.78E-13	4.34E-12	putative sigma (RP)
SCO4118	5.62E+00	1.19E-21	4.92E-20	AtraA (tetR) (RP)
SCO4174	4.82E+00	6.87E-12	1.46E-10	putative integral membrane protein(MB)
SCO4175	6.82E+00	2.27E-31	1.64E-29	hypothetical protein (HP)
SCO4214	1.11E+02	4.12E-140	3.04E-136	AbaA-like (RP)
SCO4265	4.30E+00	5.37E-13	1.27E-11	putative transport integral membrane protein MDR (MSF) MB)
SCO4266	2.02E+01	7.44E-25	3.57E-23	putative oxidoreductase (O)
SCO4280	4.93E+00	3.41E-07	3.30E-06	putative reductase (O)
SCO4291	3.52E+00	8.18E-12	1.73E-10	putative secreted protein (S)
SCO4317	3.72E+00	9.06E-15	2.43E-13	hypothetical protein (HP)
SCO4332	3.36E+00	1.24E-11	2.57E-10	putative integral membrane ATPase cation (P-ATPase) (MB)
SCO4562	4.68E+00	3.58E-09	5.41E-08	NuoA. NADH dehydrogenase subunit (O)
SCO4563	3.24E+00	8.08E-06	5.07E-05	NuoB. NADH dehydrogenase subunit (O)
SCO4578	3.21E+00	7.45E-06	4.73E-05	hypothetical protein (HP)
SCO4617	3.03E+00	2.92E-07	2.89E-06	putative ATP-binding protein (O)
SCO4622	3.53E+00	1.25E-08	1.70E-07	putative integral membrane protein (MB)
SCO4624	3.25E+00	3.52E-05	1.80E-04	hypothetical protein (HP)
SCO4640	3.91E+00	4.00E-04	1.37E-03	tetR-family transcriptional regulator (RP)
SCO4641	6.10E+00	1.39E-03	3.92E-03	putative transmembrane efflux protein (MSF- MDR) (MB)
SCO4642	1.01E+01	6.33E-04	2.02E-03	hypothetical protein (HP)
SCO4843	3.36E+00	5.38E-06	3.59E-05	putative integral membrane protein (MB)
SCO4862	3.02E+00	3.39E-11	6.65E-10	hypothetical protein (HP)
SCO4903	4.45E+00	4.31E-11	8.36E-10	putative membrane protein (MB)
SCO4983	5.01E+00	7.80E-06	4.93E-05	hypothetical protein (HP)
SCO4999	4.58E+00	1.04E-16	3.16E-15	hypothetical protein (HP)
SCO5071	8.73E+00	7.07E-29	4.43E-27	ACT cluster (SM)

SCO5072	1.69E+01	3.64E-52	5.27E-50	ACT cluster (SM)
SCO5073	1.59E+01	3.46E-59	8.25E-57	ACT cluster (SM)
SCO5074	1.52E+01	1.39E-58	3.11E-56	ACT cluster (SM)
SCO5075	7.06E+00	9.09E-34	7.55E-32	ACT cluster (SM)
SCO5078	3.45E+00	2.33E-16	6.97E-15	ACT cluster (SM)
SCO5079	4.22E+00	1.61E-25	8.37E-24	ACT cluster (SM)
SCO5080	5.23E+00	6.41E-20	2.41E-18	ACT cluster (SM)
SCO5081	4.44E+00	2.57E-18	8.70E-17	ACT cluster (SM)
SCO5085	5.86E+00	5.71E-34	4.85E-32	ACT cluster (SM). ActII-ORF4 (RP)
SCO5086	1.84E+01	8.14E-76	4.63E-73	ACT cluster (SM)
SCO5087	1.17E+01	1.85E-48	2.40E-46	ACT cluster (SM)
SCO5088	6.98E+00	6.50E-25	3.14E-23	ACT cluster (SM)
SCO5089	5.84E+00	2.55E-13	6.16E-12	ACT cluster (SM)
SCO5090	6.07E+00	2.59E-27	1.48E-25	ACT cluster (SM)
SCO5091	4.46E+00	6.74E-19	2.36E-17	ACT cluster (SM)
SCO5092	5.84E+00	2.68E-19	9.57E-18	ACT cluster (SM)
SCO5163	3.50E+00	8.07E-08	9.08E-07	hypothetical protein (HP)
SCO5345	3.39E+00	4.05E-05	2.02E-04	conserved hypothetical protein (HP)
SCO5379	3.96E+00	1.47E-14	3.85E-13	putative membrane protein (MB)
SCO5380	3.69E+00	2.76E-06	2.05E-05	putative membrane protein (MB)
SCO5551	1.53E+01	4.27E-06	2.94E-05	hypothetical protein (HP)
SCO5644	3.23E+00	1.69E-12	3.80E-11	hypothetical protein (HP)
*SCO5882**	2.53E+00	4.37E-04	1.48E-03	RED cluster (SM)
*SCO5883**	2.52E+00	4.51E-05	2.20E-04	RED cluster (SM)
*SCO5884**	1.93E+00	1.94E-03	5.22E-03	RED cluster (SM)
*SCO5888**	2.40E+00	1.28E-04	5.35E-04	RED cluster (SM)
*SCO5891**	1.70E+00	8.06E-04	2.46E-03	RED cluster (SM)

*SCO5892**	1.97E+00	3.91E-03	9.38E-03	RED cluster (SM)
SCO5924	3.12E+00	2.22E-06	1.69E-05	probable integral membrane protein iron III ABC (MB)
SCO6021	3.16E+00	2.11E-22	8.95E-21	conserved hypothetical protein (HP)
SCO6091	5.20E+00	8.06E-17	2.47E-15	putative integral membrane protein (RND) MDR
SCO6197	4.05E+00	3.15E-09	4.81E-08	putative secreted protein (S)
SCO6198	4.38E+00	2.48E-05	1.34E-04	putative secreted (S)
SCO6199	3.54E+00	1.16E-04	4.88E-04	secreted esterase (S)
SCO6228	3.79E+00	1.97E-14	5.07E-13	hypothetical protein (HP)
SCO6265	4.27E+00	2.44E-18	8.31E-17	butyrolacton ScbR (RP)
SCO6266	3.39E+01	2.15E-52	3.17E-50	butyrolacton Scb1 (SM)
SCO6267	5.59E+00	2.74E-20	1.05E-18	hypothetical protein (HP)
SCO6268	1.30E+01	7.56E-04	2.33E-03	two-component system sensor kinase HK (RP)
SCO6272	6.72E+01	4.88E-04	1.63E-03	putative secreted FAD binding (S)
SCO6273	1.12E+02	2.01E-04	7.77E-04	hexaketid cpk cluster (SM)
SCO6274	8.31E+01	1.18E-04	4.95E-04	hexaketid cpk cluster (SM)
SCO6275	1.06E+02	8.28E-05	3.70E-04	hexaketid cpk cluster (SM)
SCO6276	4.49E+02	1.30E-04	5.41E-04	hexaketid cpk cluster (SM)
SCO6277	2.31E+02	1.02E-04	4.40E-04	hexaketid cpk cluster (SM)
SCO6278	2.42E+02	1.57E-04	6.36E-04	hexaketid cpk cluster (SM)
SCO6279	2.77E+02	2.27E-04	8.55E-04	hexaketid cpk cluster (SM)
SCO6280	3.59E+01	2.26E-08	2.87E-07	hexaketid cpk cluster (SM). CpkO (RP)
SCO6281	3.82E+01	4.38E-05	2.15E-04	hexaketid cpk cluster (SM)
SCO6282	6.03E+02	1.21E-04	5.06E-04	hexaketid cpk cluster (SM)
SCO6283	4.04E+01	6.15E-04	1.97E-03	hexaketid cpk cluster (SM)
SCO6284	1.65E+01	1.74E-03	4.75E-03	hexaketid cpk cluster (SM)
SCO6285	4.55E+01	1.20E-04	5.03E-04	hexaketid cpk cluster (SM). Regulator (RP)
SCO6286	2.35E+01	5.49E-04	1.80E-03	hexaketid cpk cluster (SM)

SCO6287	2.69E+01	4.41E-04	1.49E-03	hexaketid cpk cluster (SM)	
SCO6288	2.13E+01	5.15E-04	1.70E-03	hexaketid cpk cluster (SM). Regulator (RP)	
SCO6289	4.20E+00	1.85E-03	5.00E-03	putative secreted oxidoreductase (S)	
SCO6291	3.25E+00	3.45E-04	1.22E-03	putative secreted oxidase (S)	
SCO6328	3.26E+00	1.42E-05	8.25E-05	putative membrane protein (MB)	
SCO6346	3.13E+00	9.30E-05	4.08E-04	hypothetical protein (HP)	
SCO6421	4.36E+00	4.38E-25	2.20E-23	two-component system sensor kinase HK (RP)	
SCO6422	3.78E+00	7.35E-17	2.27E-15	response regulator. NarL family (RP)	
SCO6544	5.80E+00	6.92E-31	4.92E-29	putative membrane protein (MB)	
SCO6574	3.39E+00	3.19E-05	1.66E-04	putative membrane protein (MB)	
SCO6576	3.15E+00	1.26E-06	1.04E-05	conserved hypothetical protein (HP)	
SCO6647	4.28E+00	1.34E-12	3.05E-11	putative integral membrane protein (MB)	
SCO6650	3.28E+00	1.96E-19	7.13E-18	6-pyruvoyl tetrahydropterin synthase (O)	SigU
SCO6651	5.86E+00	1.41E-29	9.07E-28	putative glycosyl transferase (O)	
SCO6652	4.60E+00	8.53E-20	3.15E-18	hypothetical protein (HP)	
SCO6653	4.71E+00	5.78E-18	1.93E-16	hypothetical protein (HP)	
SCO6749	3.26E+00	4.63E-08	5.50E-07	hypothetical protein (HP)	
SCO6773	3.13E+00	3.63E-10	6.24E-09	putative secreted peptidase (S)	
SCO6821	3.07E+00	8.89E-12	1.87E-10	putative transferase (O)	
SCO6850	3.70E+00	1.53E-06	1.23E-05	conserved hypothetical protein (HP)	
SCO6852	3.52E+00	1.87E-06	1.45E-05	conserved hypothetical protein (HP)	
SCO6858	3.11E+00	2.64E-07	2.64E-06	hypothetical protein (HP)	
SCO6872	3.12E+00	9.04E-07	7.70E-06	conserved hypothetical protein (HP)	
SCO6880	3.19E+00	1.50E-08	2.00E-07	putative lipoprotein (O)	
SCO6882	3.92E+00	3.66E-05	1.85E-04	hypothetical protein (HP)	
SCO6900	3.79E+00	2.43E-07	2.46E-06	hypothetical protein (HP)	
SCO6905	3.13E+00	1.62E-06	1.29E-05	hypothetical protein (HP)	

SCO6921	3.03E+00	3.28E-11	6.44E-10	putative membrane protein (MB)
SCO6924	3.11E+00	3.32E-07	3.24E-06	putative DNA-binding protein (RP)
SCO6957	3.02E+00	2.24E-05	1.22E-04	putative integral membrane protein (CPA3 family)(MB)
SCO6974	3.25E+00	1.64E-10	2.95E-09	tetR-family transcriptional regulator (RP)
SCO6977	3.19E+00	2.73E-09	4.19E-08	hypothetical protein (HP)
SCO7008	3.12E+00	2.35E-06	1.78E-05	putative ABC-transport protein MDR (MB)
SCO7034	3.11E+00	5.38E-08	6.29E-07	putative aminotransferase (fragment) (O)
SCO7066	7.30E+00	1.85E-03	5.00E-03	2.4 dienoil-CoA reductase (NADPH) (O)
SCO7082	3.08E+00	1.58E-05	9.06E-05	hypothetical protein (HP)
SCO7098	3.05E+00	1.01E-06	8.50E-06	conserved hypothetical protein (HP)
SCO7106	3.30E+00	7.49E-07	6.60E-06	conserved hypothetical protein (HP)
SCO7113	3.71E+00	4.76E-07	4.44E-06	putative integral membrane protein (MB)
SCO7114	3.02E+00	1.66E-06	1.32E-05	conserved hypothetical protein (HP)
SCO7122	3.39E+00	1.49E-06	1.20E-05	putative acetyltransferase (O)
SCO7125	3.01E+00	2.98E-06	2.18E-05	conserved hypothetical protein (HP)
SCO7152	4.21E+00	1.66E-09	2.62E-08	hypothetical protein (HP)
SCO7160	3.03E+00	1.58E-06	1.26E-05	conserved hypothetical protein (HP)
SCO7165	3.24E+00	2.77E-06	2.05E-05	sugar-binding integral membrane transport protein ABC (MB)
SCO7192	4.30E+00	1.44E-03	4.04E-03	put sigma (RP)
SCO7225	3.98E+00	3.87E-10	6.57E-09	secreted chitinase (S)
SCO7273	3.21E+00	2.76E-05	1.46E-04	hypothetical protein (HP)
SCO7345	3.19E+00	3.09E-08	3.79E-07	probable ATP-dependent DNA ligase (O)
SCO7450	3.82E+00	7.89E-06	4.97E-05	putative secreted protein (S)
SCO7488	3.62E+00	1.77E-06	1.39E-05	putative integral membrane ABC binding protein (MB)
SCO7489	3.12E+00	1.18E-10	2.18E-09	putative binding protein dependent transport lipoprotein (MB)
SCO7513	3.28E+00	2.81E-05	1.48E-04	putative secreted hydrolase (S)
SCO7536	1.38E+01	4.13E-18	1.39E-16	putative integral membrane protein (RND) (MB)

SCO7577	3.67E+00	9.24E-09	1.30E-07	putative secreted hydrolase (S)
SCO7638	5.83E+00	3.24E-20	1.22E-18	enolase (O)
SCO7669	3.08E+00	2.52E-05	1.35E-04	aromatic polyketide (SM)
SCO7671	3.14E+00	2.93E-03	7.40E-03	aromatic polyketide (SM)
SCO7717	5.95E+00	6.42E-10	1.07E-08	putative secreted protein (S)
SCO7759	3.44E+00	3.45E-07	3.33E-06	putative DNA-binding protein (RP)
SCO7761	3.88E+00	7.89E-06	4.97E-05	hypothetical protein (HP)
SCO7762	4.46E+00	3.21E-06	2.32E-05	hypothetical protein (HP)

1 Gordon. N. D. *et al.* Secreted-protein response to sigmaU activity in *Streptomyces coelicolor*. *J Bacteriol* **190**. 894-904 (2008).
