# Supplementary material for: The Orphan Response Regulator Aor1 Is a New Relevant Piece in the Complex Puzzle of Streptomyces coelicolor Antibiotic Regulatory Network
Source: Front Microbiol. 2017 Dec 12;8:2444. doi: 10.3389/fmicb.2017.02444 (PMC5733086; doi:10.3389/fmicb.2017.02444)
Supplement: Supplementary file 4 [file Table_4.PDF]

Supplementary Table S4: *Genes differentially up-regulated in ∆aor1 (RNA-seq)*

Genes whose transcript levels changed at least 3-fold (FC) *in S. coelicolor* ∆*aor1* compared with those of M145 (p value ≤ 0.002 and FDR ≤ 0.01).

* FC: relevant genes with FC<3. Secondary metabolites: SM; Regulatory proteins: RP; Membrane proteins: MB; Gas vesicle proteins: GV; Hypothetical proteins: HP; Secreted proteins: S; Other functions: O.

Gene	FC	p-value	FDR	Function	Regulon [1-4]
SCO0072	5.79E+00	2.60E-30	1.76E-28	putative secreted protein (S)	
SCO0103	4.52E+00	5.00E-25	2.47E-23	putative flavohemoprotein (O)	
SCO0104	3.39E+00	3.84E-12	8.38E-11	putative hydrolase (O)	
SCO0130	5.33E+00	2.32E-43	2.81E-41	putative beta-lactamase (O)	
SCO0197	3.42E+00	3.48E-09	5.29E-08	conserved hypothetical protein (HP)	
SCO0212	3.62E+00	2.46E-09	3.78E-08	hypothetical protein (HP)	
SCO0217	3.93E+00	2.90E-08	3.60E-07	nitrate reductase beta chain NarH2 (O)	
SCO0218	4.84E+00	7.73E-10	1.28E-08	putative nitrate reductase delta chain NarJ2 (O)	
SCO0219	3.94E+00	6.10E-08	7.03E-07	putative nitrate reductase delta chain NarI2 (O)	
SCO0220	4.40E+00	3.46E-09	5.27E-08	hypothetical protein (HP)	
SCO0231	2.34E+01	7.32E-65	2.71E-62	small hydrophobic hypothetical protein (HP)	
SCO0256	8.41E+00	1.13E-05	6.80E-05	putative short chain oxidoreductase (O)	
SCO0257	1.04E+01	2.41E-06	1.81E-05	conserved hypothetical protein (HP)	
SCO0258	7.43E+00	2.01E-05	1.11E-04	hypothetical protein (HP)	
SCO0259	8.56E+00	5.24E-06	3.51E-05	putative alcohol dehydrogenase (zinc-binding) (O)	
SCO0260	3.40E+00	2.14E-07	2.20E-06	conserved hypothetical protein (HP)	
SCO0595	4.32E+00	2.06E-24	9.58E-23	conserved hypothetical protein (HP)	
SCO0596	2.61E+01	2.00E-62	5.69E-60	DNA binding (RP)	
*SCO0596**	2.61E+01	2.00E-62	5.69E-60	DpsA (sigB reg) (O)	
SCO0600	5.67E+00	3.82E-27	2.16E-25	SigB (RP)	SigB

SCO0666	1.14E+01	6.45E-34	5.42E-32	CatB (sigB reg) (O)	SigB
SCO0678	2.81E+01	5.98E-79	4.91E-76	hypothetical protein (HP)	
SCO0681	3.35E+00	1.90E-08	2.47E-07	putative ferredoxin/ferredoxin-NADP reductase secreted protein (S)	
SCO0686	4.24E+00	1.10E-24	5.24E-23	putative membrane protein (MB)	
SCO0689	4.59E+00	2.74E-28	1.66E-26	putative oxidoreductase. iron-sulphur binding subunit (O)	
SCO0698	6.74E+00	1.04E-43	1.28E-41	hypothetical protein (HP)	
SCO0718	4.93E+00	3.50E-29	2.21E-27	hypothetical protein (HP)	
SCO0719	3.76E+00	2.82E-23	1.25E-21	conserved hypothetical protein (HP)	
SCO0721	4.78E+00	7.12E-24	3.25E-22	putative glycosyl transferase (O)	
SCO0722	5.77E+00	4.35E-33	3.46E-31	putative hydrolase (O)	
SCO0750	1.14E+01	4.06E-66	1.87E-63	hypothetical protein (HP)	
SCO0753	4.73E+00	2.31E-27	1.33E-25	bacteriocin (SM)	
SCO0757	3.57E+00	1.06E-19	3.86E-18	putative phosphatase (O)	
SCO0758	4.39E+00	8.76E-19	3.04E-17	hypothetical protein (HP)	
SCO0759	6.21E+00	6.02E-28	3.53E-26	hypothetical protein (HP)	SigB
SCO0760	4.11E+00	4.31E-21	1.75E-19	putative methyltransferase (O)	
SCO0761	3.36E+00	2.35E-06	1.78E-05	hypothetical protein (HP)	
*SCO0762**	2.50E+00	2.06E-06	1.58E-05	protease inhibitor Sti1	SigU
SCO0776	5.41E+00	6.35E-45	7.96E-43	putative integral membrane protein cation efflux (CDF family) (MB)	
SCO0777	4.59E+00	4.46E-35	3.97E-33	conserved hypothetical protein (HP)	
SCO0778	3.33E+00	1.22E-17	3.99E-16	putative integral membrane protein (MB)	
SCO0779	1.26E+01	3.53E-54	6.07E-52	conserved hypothetical protein (HP)	
SCO0784	9.80E+00	2.76E-60	6.81E-58	secreted protein (S)	
SCO0786	3.79E+00	6.49E-26	3.45E-24	putative integral membrane protein (MB)	
SCO0790	6.33E+00	7.87E-13	1.84E-11	putative hydrolase (O)	
SCO0792	3.05E+00	1.40E-14	3.70E-13	hypothetical protein (HP)	SigB
SCO0793	4.01E+00	1.56E-14	4.07E-13	hypothetical protein (HP)	

SCO0827	3.84E+00	6.62E-12	1.42E-10	hypothetical protein (HP)
SCO0834	3.46E+00	1.91E-16	5.78E-15	hypothetical protein (HP)
SCO0844	3.08E+00	7.41E-11	1.41E-09	possible DNA-binding protein (RP)
SCO0845	4.77E+00	6.06E-16	1.74E-14	hypothetical protein (HP)
SCO0870	3.22E+00	3.25E-15	8.90E-14	putative two-component system response regulator (RP)
SCO0871	3.17E+00	9.54E-15	2.54E-13	putative two-component sensor protein (RP)
SCO0885	4.71E+00	8.57E-27	4.76E-25	trxC (O)
SCO0896	6.64E+00	4.41E-35	3.97E-33	putative oxidoreductase (O)
SCO0897	1.06E+01	5.21E-58	1.13E-55	conserved hypothetical protein (HP)
SCO0906	4.21E+00	4.55E-25	2.27E-23	hypothetical protein (HP)
SCO0919	3.30E+00	8.05E-17	2.47E-15	hypothetical protein (HP)
SCO1124	3.60E+00	1.68E-09	2.65E-08	putative integral membrane protein (MB)
SCO1125	3.08E+00	3.52E-13	8.42E-12	hypothetical protein (HP)
SCO1130	3.50E+00	8.55E-11	1.61E-09	conserved hypothetical protein (HP)
SCO1174	3.43E+00	1.73E-04	6.86E-04	aldehyde dehydrogenase (O)
SCO1204	3.64E+00	1.61E-21	6.63E-20	putative aldehyde dehydrogenase(O)
SCO1223	3.27E+00	2.23E-07	2.28E-06	ornithine aminotransferase (O)
SCO1225	9.56E+00	1.64E-55	2.95E-53	putative osmoprotectant transporter proline MSF (MB)
SCO1491	3.71E+00	1.72E-10	3.06E-09	elongation factor P(O)
SCO1492	3.60E+00	1.26E-14	3.35E-13	putative peptidase (O)
SCO1557	7.92E+00	4.32E-15	1.18E-13	putative lipoprotein (MB)
*SCO1864**	1.85E+00	7.24E-07	6.41E-06	5-hydroxiectoin cluster (SM)
*SCO1865**	1.69E+00	6.64E-04	2.10E-03	5-hydroxiectoin cluster (SM)
*SCO1866**	2.25E+00	8.83E-06	5.47E-05	5-hydroxiectoin cluster (SM)
*SCO1867**	2.03E+00	1.23E-04	5.15E-04	5-hydroxiectoin cluster (SM)
SCO1898	8.99E+00	2.79E-08	3.47E-07	substrate binding ABC (MB)
SCO1899	9.33E+00	3.13E-08	3.84E-07	sugar transport ABC (MB)

SCO1900	1.55E+01	2.07E-10	3.64E-09	sugar transport ABC (MB)	
SCO1901	1.68E+01	7.27E-12	1.54E-10	zinc binding deydrogenase (O)	
SCO1902	6.44E+00	3.22E-07	3.14E-06	hydrophilic protein (O)	
SCO1910	1.26E+01	1.29E-51	1.84E-49	putative alanine-rich protein (O)	
SCO1911	1.28E+01	3.73E-54	6.26E-52	putative integral membrane protein (MB)	
SCO2113	3.35E+00	2.22E-04	8.41E-04	bacterioferritin (O)	
SCO2136	3.68E+00	3.87E-12	8.40E-11	putative secreted protein (S)	
SCO2210	5.05E+00	6.02E-12	1.29E-10	glutamine synthetase (O)	
SCO2280	3.94E+00	5.49E-25	2.67E-23	putative TetR-family transcriptional regulator (RP)	
SCO2315	1.05E+01	3.43E-43	4.09E-41	putative membrane protein (MB)	
SCO2341	1.54E+01	5.86E-56	1.14E-53	hypothetical protein (HP)	
SCO2342	1.73E+01	7.34E-53	1.15E-50	putative secreted protein (S)	
SCO2344	3.15E+00	6.42E-17	2.01E-15	putative integral membrane transport protein MDR (MB)	
SCO2372	7.20E+00	2.74E-46	3.49E-44	hydrophobic (O)	SigB
SCO2453	1.34E+01	1.14E-61	3.01E-59	secreted proein (S)	
SCO2493	4.87E+00	1.26E-22	5.43E-21	putative membrane protein (MB)	
SCO2494	4.82E+00	1.31E-08	1.78E-07	putative pyruvate phosphate dikinase (O)	
SCO2628	3.93E+00	4.80E-12	1.04E-10	putative amino acid permease (O)	
SCO2788	3.08E+00	2.01E-08	2.60E-07	hypothetical protein (HP)	
SCO2789	3.06E+00	1.88E-07	1.95E-06	glucosamine-fructose-6-phosphate aminotransferase (O)	
SCO2886	5.69E+00	1.65E-33	1.33E-31	putative hydrolase (O)	
SCO2907	3.32E+00	1.56E-15	4.31E-14	putative PTS transmembrane component (NAG IIA) (MB)	
SCO2910	3.34E+00	9.57E-10	1.56E-08	putative cysteine synthase (O)	
SCO2911	3.05E+00	9.27E-10	1.52E-08	conserved hypothetical protein (HP)	
SCO2924	3.79E+00	4.81E-25	2.39E-23	putative regulator (RP)	
*SCO3034**	2.68E+00	6.56E-08	7.51E-07	whiB (O)	
SCO3064	3.44E+00	4.16E-10	7.00E-09	putative peptide transporter (MB)	

SCO3139	3.18E+00	8.80E-17	2.68E-15	putative sodium:solute symporter (O)	
SCO3158	4.34E+00	4.99E-21	2.02E-19	hypothetical protein (HP)	
*SCO3226**	1.80E+00	6.59E-06	4.25E-05	response regulator AbsA2 (RP)	
SCO3311	3.08E+00	1.51E-08	2.02E-07	delta-aminolevulinic acid dehydratase (O)	
SCO3802	1.26E+01	2.14E-55	3.77E-53	putative membrane protein (MB)	
SCO3805	4.10E+00	2.62E-25	1.34E-23	hypothetical protein (HP)	
SCO3857	3.50E+00	1.41E-15	3.93E-14	putative regulatory protein (RP)	
SCO4029	7.17E+00	3.96E-38	3.91E-36	hypothetical protein (HP)	
SCO4077	3.13E+00	1.84E-08	2.41E-07	hypothetical protein (HP)	
SCO4078	4.64E+00	2.51E-11	5.03E-10	phosphoribosyl formylglycinamidine synthase I (O)	
SCO4079	4.36E+00	2.08E-09	3.25E-08	phosphoribosyl formylglycinamidine synthase II (O)	
SCO4164	7.07E+00	9.21E-18	3.05E-16	putative thiosulfate sulfurtransferase (O)	
SCO4165	5.34E+00	3.94E-19	1.39E-17	conserved hypothetical protein (HP)	
SCO4498	1.56E+01	3.21E-42	3.71E-40	proton/sodium ion:glutamate transport (DAACS family) (MB)	
SCO4734	3.42E+00	1.73E-08	2.27E-07	50S L13 (O)	
SCO4799	3.22E+00	2.69E-09	4.13E-08	putative secreted lipase (S)	
SCO4829	3.28E+00	2.06E-13	5.00E-12	putative oxidoreductase (O)	
SCO4846	3.26E+00	9.16E-12	1.91E-10	putative integral membrane protein (MB)	
SCO4896	3.14E+00	8.01E-10	1.32E-08	putative transport integral membrane protein (MSF) MDR (MB)	
SCO4982	4.68E+00	6.92E-27	3.87E-25	putative membrane protein (MB)	
SCO5178	3.96E+00	4.71E-11	9.06E-10	putative sulfurylase (O)	
SCO5243	1.26E+01	1.60E-41	1.80E-39	sig H (RP)	
SCO5244	1.14E+01	2.46E-41	2.67E-39	anti sigma H (RP)	SigB
SCO5529	3.42E+00	1.34E-15	3.76E-14	putative 2-isopropylmalate synthase (O)	
SCO5650	3.07E+00	5.08E-09	7.41E-08	putative membrane protein (MB)	
SCO5966	6.98E+00	2.07E-26	1.12E-24	putative oxidase (O)	
SCO5982	3.40E+00	1.09E-13	2.67E-12	putative regulator (RP)	

SCO6014	3.46E+00	1.97E-19	7.14E-18	putative cationic amino acid transporter (APC family) (MB)	
SCO6051	5.95E+00	4.20E-30	2.80E-28	putative iron-sulfur binding oxidoreductase (O)	
SCO6141	7.71E+00	1.28E-34	1.11E-32	putative transmembrane protein (MB)	
SCO6257	3.36E+00	8.23E-14	2.04E-12	putative ABC transport system sugar binding lipoprotein (MB)	
SCO6258	3.34E+00	2.75E-19	9.78E-18	putative ABC transport system sugar permease (MB)	
SCO6259	3.41E+00	1.69E-17	5.49E-16	probable ABC sugar transport ATP binding protein (MB)	
SCO6494	7.22E+00	3.75E-36	3.43E-34	putative membrane protein (MB)	SigB
SCO6495	4.61E+00	3.32E-30	2.23E-28	putative dehydrogenase (O)	
SCO6496	5.52E+00	5.32E-28	3.20E-26	gas vesicle protein (GV)	SigB
SCO6497	5.83E+00	1.07E-27	6.25E-26	gas vesicle protein (GV)	SigB
SCO6499	2.45E+01	3.61E-65	1.48E-62	gas vesicle protein (GV)	SigB
SCO6500	2.25E+01	3.84E-74	2.03E-71	gas vesicle protein (GV)	SigB
SCO6501	2.29E+01	4.26E-56	8.50E-54	gas vesicle protein (GV)	SigB
SCO6502	3.33E+01	2.17E-92	4.02E-89	gas vesicle protein (GV)	SigB
SCO6503	1.63E+01	8.68E-68	4.28E-65	gas vesicle protein (GV)	SigB
SCO6504	2.21E+01	2.28E-77	1.53E-74	gas vesicle protein (GV)	SigB
SCO6505	1.94E+01	7.61E-77	4.69E-74	gas vesicle protein (GV)	SigB
SCO6506	1.18E+01	4.43E-63	1.42E-60	gas vesicle protein (GV)	SigB
SCO6507	1.61E+01	3.18E-29	2.03E-27	gas vesicle protein (GV)	SigB
SCO6508	1.41E+01	1.96E-50	2.68E-48	gas vesicle protein (GV)	SigB
SCO6509	1.29E+01	7.44E-54	1.19E-51	hydrophobic protein (O)	
SCO6510	5.74E+00	1.81E-31	1.34E-29	conserved hypothetical protein (HP)	
SCO6511	9.73E+00	6.27E-61	1.60E-58	conserved hypothetical protein (HP)	
SCO6513	8.02E+00	7.74E-31	5.45E-29	hypothetical protein (HP)	
SCO6514	1.01E+01	1.39E-52	2.10E-50	hypothetical protein (HP)	
SCO6515	2.70E+01	1.51E-80	1.60E-77	putative protease (O)	
SCO6516	2.92E+01	1.36E-79	1.25E-76	hypothetical protein (HP)	SigB

SCO6520	6.63E+00	5.46E-35	4.81E-33	sigK (RP)	
SCO6682	4.96E+00	8.58E-11	1.61E-09	lant (sapB) (SM) RR (RP)	
SCO6685	3.50E+00	5.09E-14	1.28E-12	lantibiotic (sapB) (SM)	
SCO6706	9.34E+00	4.64E-40	4.89E-38	putative glutathione-dependent aldehyde dehydrogenase (O)	
SCO6724	3.15E+00	4.26E-17	1.35E-15	hypothetical protein (HP)	
SCO6965	4.00E+00	2.48E-20	9.61E-19	hypothetical protein (HP)	
SCO7040	3.11E+00	2.35E-07	2.38E-06	glyceraldehyde-3-phosphate dehydrogenase (O)	
SCO7041	1.13E+01	2.28E-36	2.13E-34	hypothetical protein (HP)	SigB
SCO7186	7.57E+00	9.73E-51	1.36E-48	putative integral membrane protein (MB)	
SCO7210	2.56E+01	7.96E-78	5.88E-75	conserved hypothetical protein (HP)	
SCO7236	1.22E+01	2.99E-62	8.20E-60	ubiquinol-cytochrome C reductase cytochrome subunit B (O)	
SCO7237	1.93E+01	7.09E-83	8.73E-80	secreted protein (S)	SigB
SCO7238	1.16E+01	8.52E-56	1.57E-53	hypothetical protein (HP)	SigB
SCO7260	7.44E+00	8.88E-40	9.25E-38	possible membrane protein (MB)	
SCO7276	4.44E+00	5.46E-28	3.26E-26	hypothetical protein (HP)	
SCO7277	3.58E+00	6.31E-23	2.73E-21	putative regulator protein (RP)	SigB
SCO7278	6.68E+00	7.09E-32	5.30E-30	SigL (RP)	SigB
SCO7287	9.09E+00	1.29E-30	8.94E-29	hypothetical protein (HP)	
SCO7288	3.95E+00	2.24E-26	1.21E-24	possible membrane protein (MB)	
SCO7289	8.40E+00	1.72E-41	1.89E-39	ssgC (O)	SigB
SCO7290	5.93E+00	2.05E-39	2.10E-37	putative dehydrogenase (O)	
SCO7305	3.59E+00	1.94E-22	8.31E-21	conserved hypothetical protein (HP)	
SCO7306	3.96E+00	2.78E-14	7.10E-13	regulatory protein (RP)	
SCO7311	9.26E+00	3.41E-50	4.58E-48	probable amino acid decarboxylase (O)	
SCO7312	5.95E+00	9.37E-34	7.70E-32	hypothetical protein (HP)	
SCO7314	3.17E+01	5.29E-86	7.82E-83	SigM (RP)	
SCO7315	1.14E+01	2.26E-23	1.01E-21	hypothetical protein (HP)	

SCO7316	1.25E+01	1.37E-52	2.10E-50	putative integral membrane protein (MB)	
SCO7317	2.06E+01	5.68E-96	1.40E-92	hypothetical protein (HP)	
SCO7325	1.47E+01	1.91E-64	6.72E-62	RsbV anti-anti sigma B (RP)	SigB
SCO7330	3.76E+00	6.54E-07	5.86E-06	putative membrane protein (MB)	
SCO7331	7.53E+00	2.15E-57	4.41E-55	conserved hypothetical protein (HP)	
SCO7371	1.71E+01	1.81E-57	3.83E-55	hypothetical protein (HP)	
SCO7373	3.05E+00	7.07E-06	4.53E-05	hypothetical protein (HP)	
SCO7374	6.25E+00	9.66E-18	3.19E-16	nitrate reductase NarB (fragment) (O)	SigB
SCO7376	4.94E+00	5.72E-28	3.38E-26	conserved hypothetical protein (HP)	
SCO7377	5.67E+00	1.41E-15	3.93E-14	hypothetical protein (HP)	
SCO7378	9.34E+00	1.65E-23	7.41E-22	hypothetical protein (HP)	SigB
SCO7379	3.35E+00	1.09E-09	1.77E-08	hypothetical protein (HP)	
SCO7381	4.82E+00	2.24E-17	7.17E-16	hypothetical protein (HP)	
SCO7387	4.41E+00	7.15E-20	2.67E-18	hypothetical protein (HP)	
SCO7388	3.17E+00	1.96E-08	2.54E-07	putative dehydrogenase (O)	
SCO7393	1.65E+01	6.79E-54	1.12E-51	putative lipoprotein (MB)	
SCO7403	3.95E+00	3.42E-15	9.32E-14	putative membrane protein (MB)	
SCO7412	2.26E+01	8.17E-59	1.89E-56	putative pyruvate oxidase (O)	
SCO7413	8.83E+00	3.05E-37	2.96E-35	putative oxidoreductase (O)	
SCO7416	4.04E+00	4.03E-23	1.76E-21	hypothetical protein (HP)	
SCO7417	4.23E+00	2.01E-20	7.91E-19	putative cytochrome P450-family protein (O)	
SCO7418	3.83E+00	3.06E-20	1.17E-18	putative cytochrome P450-family protein (O)	
SCO7419	5.09E+00	1.13E-23	5.14E-22	putative ATP/GTP binding protein (O)	
SCO7420	4.48E+00	2.34E-19	8.45E-18	hypothetical protein (HP)	
SCO7421	3.34E+00	6.63E-18	2.21E-16	conserved hypothetical protein (HP)	
SCO7423	4.01E+00	1.10E-17	3.63E-16	conserved hypothetical protein (HP)	
SCO7428	3.62E+00	4.18E-11	8.14E-10	flavohemoprotein (O)	

SCO7431	9.54E+00	7.51E-56	1.42E-53	putative integral membrane protein (MB)	SigB
SCO7434	7.77E+00	2.22E-31	1.62E-29	putative lipoprotein (MB)	
SCO7442	8.88E+00	2.46E-38	2.45E-36	hypothetical protein (HP)	
SCO7446	8.06E+00	1.01E-62	2.99E-60	putative regulator (RP)	
SCO7454	4.66E+00	2.72E-16	8.07E-15	putative membrane protein (MB)	
SCO7463	3.47E+00	2.58E-10	4.46E-09	putative sensor histidine kinase (RP)	
SCO7464	3.69E+00	2.01E-12	4.51E-11	hypothetical protein (HP)	
SCO7465	3.50E+00	1.30E-12	2.96E-11	hypothetical protein (HP)	
SCO7466	3.47E+00	2.11E-11	4.28E-10	putative regulator (RP)	
SCO7494	6.54E+00	6.88E-24	3.16E-22	putative membrane protein (MB)	
SCO7590	6.86E+00	8.99E-33	6.99E-31	catalase (O)	
SCO7646	1.46E+01	5.78E-63	1.78E-60	putative membrane protein (MB)	
SCO7747	1.26E+01	4.31E-66	1.88E-63	conserved hypothetical protein (HP)	
SCO7748	3.03E+00	3.28E-16	9.60E-15	conserved hypothetical protein (HP)	
SCO7751	3.83E+00	3.34E-25	1.69E-23	putative regulatory protein (RP)	
SCO7821	1.10E+01	8.14E-42	9.26E-40	putative integral membrane protein (MB)	

1 Facey. P. D. *et al.* The *dpsA* gene of *Streptomyces coelicolor:* induction of expression from a single promoter in response to environmental stress or during development. *PloS one* **6**. e25593. doi:10.1371/journal.pone.0025593 (2011).

2 Lee. E. J.. Cho. Y. H.. Kim. H. S. & Roe. J. H. Identification of sigmaB-dependent promoters using consensus-directed search of *Streptomyces coelicolor* genome. *Journal of microbiology* **42**. 147-151 (2004).

3 Lee. E. J. *et al.* A master regulator sigmaB governs osmotic and oxidative response as well as differentiation via a network of sigma factors in *Streptomyces coelicolor*. *Mol Microbiol* **57**. 1252-1264 (2005).

4 Gordon. N. D. *et al.* Secreted-protein response to sigmaU activity in *Streptomyces coelicolor*. *J Bacteriol* **190**. 894-904 (2008).
