# Supplementary material for: The Orphan Response Regulator Aor1 Is a New Relevant Piece in the Complex Puzzle of Streptomyces coelicolor Antibiotic Regulatory Network
Source: Front Microbiol. 2017 Dec 12;8:2444. doi: 10.3389/fmicb.2017.02444 (PMC5733086; doi:10.3389/fmicb.2017.02444)
Supplement: Supplementary file 5 [file Table_5.PDF]

Supplementary Table S5: ***q-RT-PCR of selected genes differentially expressed in the RNA-Seq assay.*** Three genes down-regulated (*cda**: *SCO3230*; *actII-ORF4: SCO5085; cpkO: SCO6280*) and three up-regulated (*dspA*: *SCO0596*; *gvp***: *SCO6502*; *sigH*: *SCO5243*) were chosen to perform qRT-PCR and validate the RNA-seq data trend of the *S. coelicolor* Δ*aor1* compared with M145. The Relative mRNA expression and Standard error data correspond to the mean of the measure of biological triplicates plus technical triplicates.

	**M145**		***Δaor1***		
Gene	Relative mRNA expresion	Standard Error	Relative mRNA expresion	Standard Error	Trend
cda*	0,950	0,338	0,437	0,016	DOWNREGULATED
actII-ORF4	1,051	0,376	0,530	0,058	DOWNREGULATED
cpkO	0,795	0,246	0,308	0,003	DOWNREGULATED
dpsA	1,000	0,317	2,327	0,261	UPREGULATED
gvp**	0,921	0,394	1,618	0,223	UPREGULATED
sigH	1,490	0,622	2,585	0,115	UPREGULATED
