# Supplementary figures and images for: The Orphan Response Regulator Aor1 Is a New Relevant Piece in the Complex Puzzle of Streptomyces coelicolor Antibiotic Regulatory Network
Source: Front Microbiol. 2017 Dec 12;8:2444. doi: 10.3389/fmicb.2017.02444 (PMC5733086; doi:10.3389/fmicb.2017.02444)

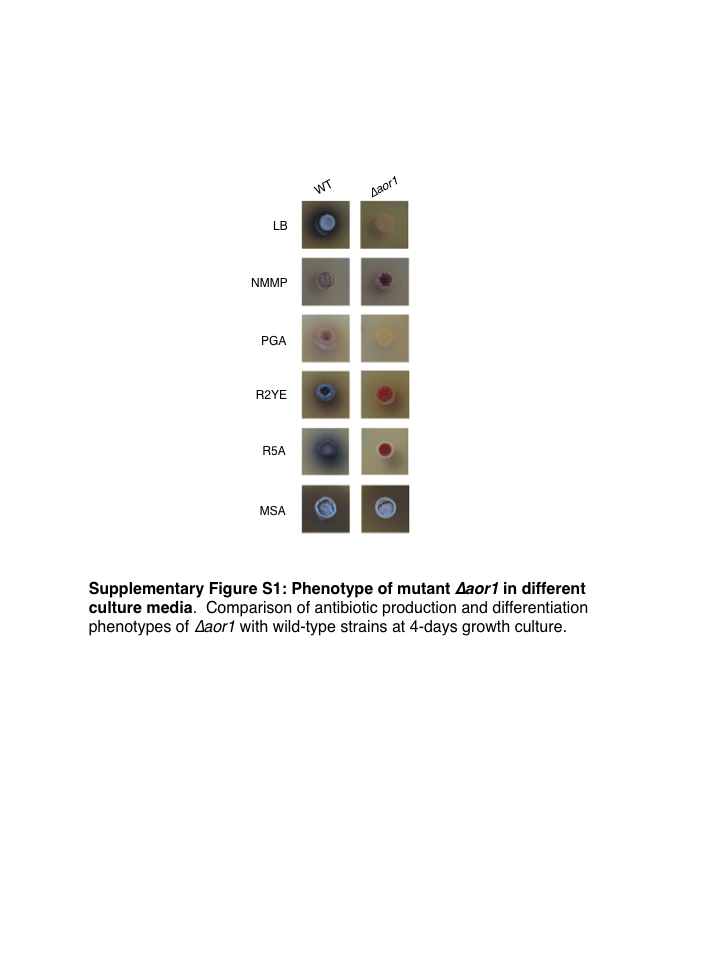

Supplement: Supplementary file 6 [file Image_1.TIF]

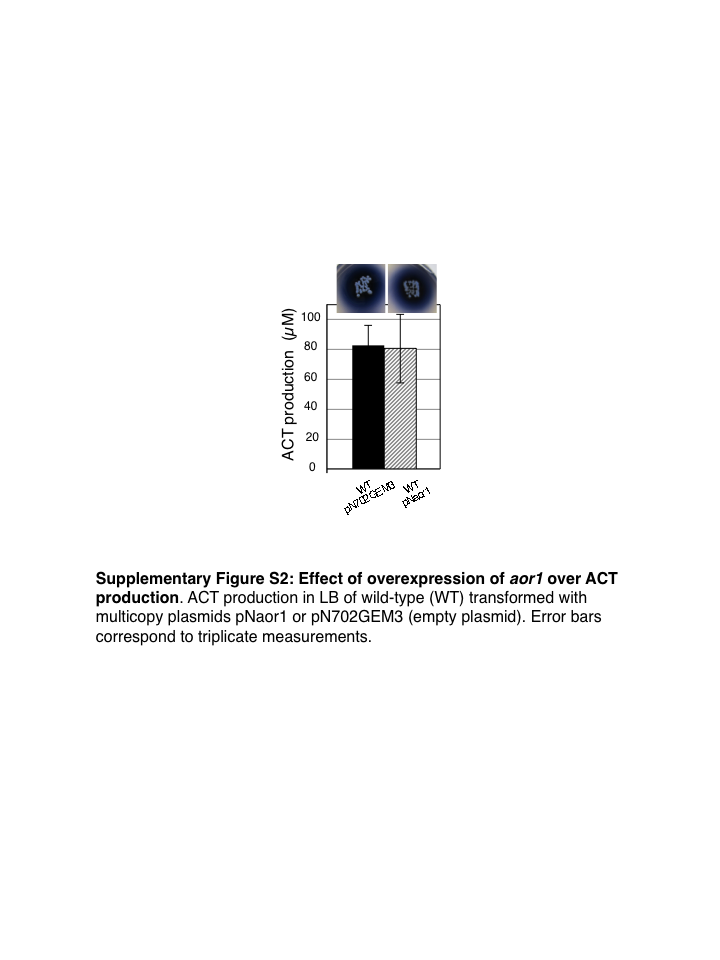

Supplement: Supplementary file 7 [file Image_2.TIF]

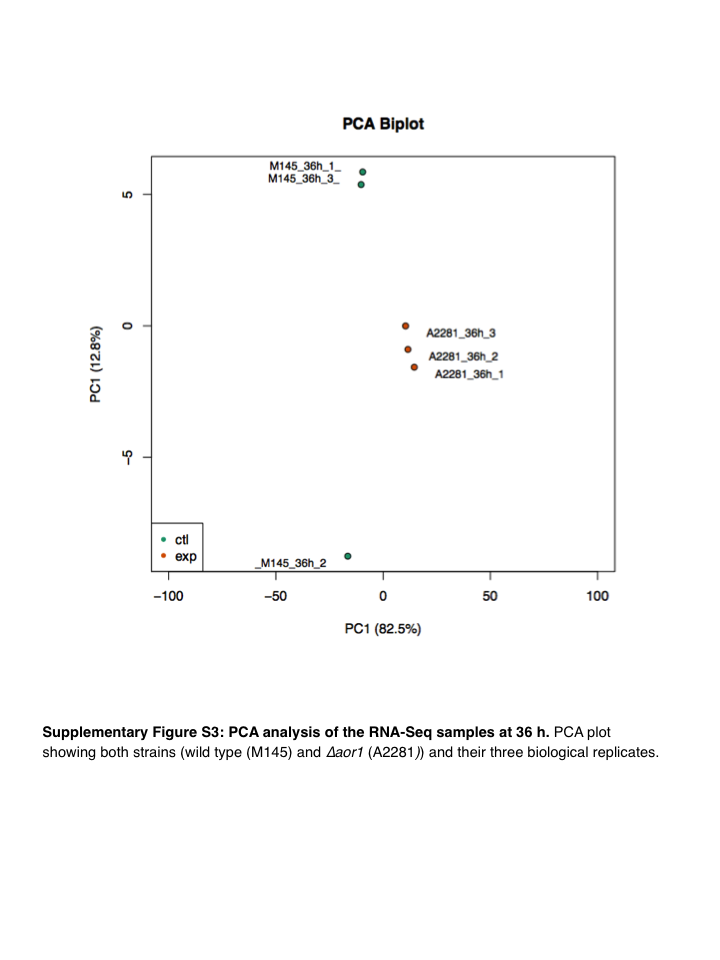

Supplement: Supplementary file 8 [file Image_3.TIF]

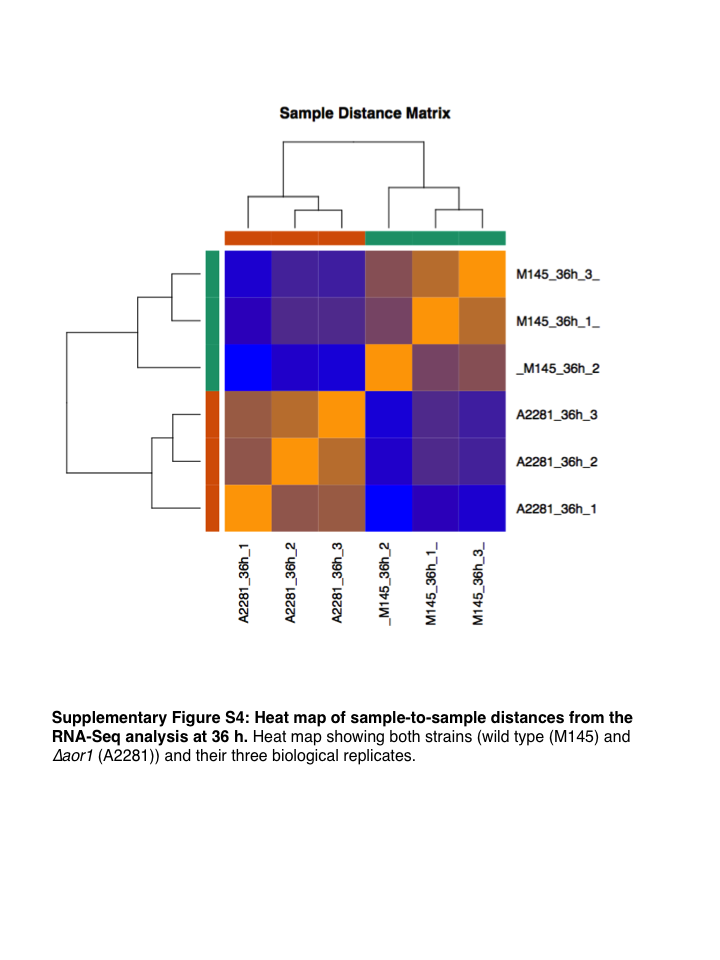

Supplement: Supplementary file 9 [file Image_4.TIF]

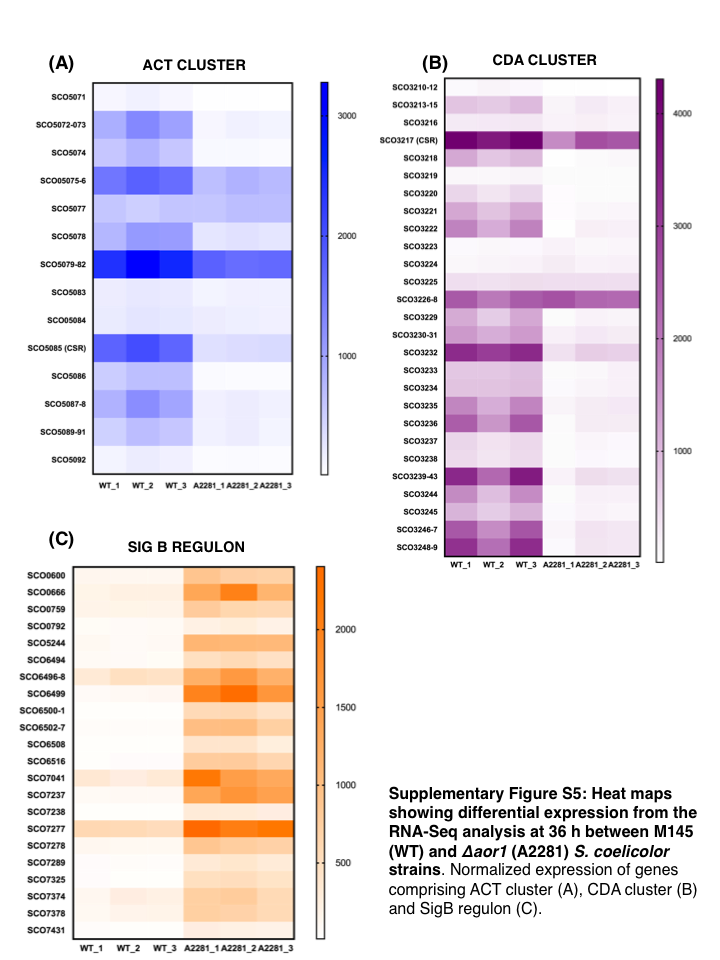

Supplement: Supplementary file 10 [file Image_5.TIF]
